# New Histone H4 variant and H2B variant Exhibits distinct genomic distributions, chromatin affinities, and dynamics throughout life and cell cycle of *Trypanosoma cruzi*

**DOI:** 10.1371/journal.ppat.1013310

**Published:** 2026-03-31

**Authors:** Juliana Nunes Roson, Mariana Loterio-Silva, Herbert Guimaraes de Sousaes Silva, Thaina Rodrigues de Almeida, Nadjania Saraiva de Lira Silva, Camila Gachet-Castro, Héllida Marina Costa-Silva, Vincent Louis Viala, Sergio Schenkman, M. Carolina Elias, Julia P.C. da Cunha

**Affiliations:** 1 Laboratory of Cell Cycle, Butantan Institute, São Paulo, Brazil; 2 Center of Toxins, Immune Response and Cell Signaling (CeTICS), Butantan Institute, São Paulo, Brazil; 3 Department of Microbiology, Immunology, and Parasitology, Escola Paulista de Medicina – UNIFESP, São Paulo, Brazil; 4 ARIES, Antimicrobial Research Institute of São Paulo, Escola Paulista de Medicina – UNIFESP, São Paulo, Brazil; 5 Biochemistry Laboratory, Butantan, Institute, São Paulo, Brazil; University of Texas Southwestern Medical School, UNITED STATES OF AMERICA

## Abstract

Histone variants play crucial roles in chromatin organization and transcriptional regulation in eukaryotes. Unusually, trypanosomatids display histone variants for all histones, although a functional homolog of histone H4 variant (H4.V) had not yet been described in *Trypanosoma cruzi*. In this study, we identified a H4.V in *T. cruzi* that is encoded by a single-copy gene located apart from the typical tandem arrays of canonical histone H4. Functional characterization using ChIP-seq assays revealed that H4.V is located at telomere-subtelomere interface, demarcates convergent strand-switch regions (cSSRs), and determines potential new transcription termination sites at codirectional PTUs interrupted by tDNA loci. Throughout the cell cycle, H4.V abundance increases in G2/M, as shown by immunofluorescence and imaging flow cytometry. In contrast, the histone H2B variant (H2B.V) abundance accumulates progressively. H4.V is more abundant in the nuclei of metacyclic trypomastigotes but barely detectable in amastigotes and bloodstream trypomastigotes. In contrast, H2B.V shows a punctate nuclear pattern and is present in all life stages, with the highest levels also observed in metacyclics. During metacyclogenesis, both variants show a progressive increase in expression, particularly H4.V, suggesting a role in parasite differentiation. Ultimately, salt extraction experiments revealed life stage–dependent differences in histone distribution profiles, particularly between insect-stage and mammalian-stage parasites, with H4.V displaying greater retention in the salt-resistant chromatin fraction, whereas H2B.V was more readily released under low-salt conditions compared to canonical histone H3. Our data reveal H4.V as a novel histone variant in *T. cruzi*, characterized by unique genomic localization, expression profiles, and chromatin-binding dynamics in contrast to H2B.V. These findings underscore H4.V as a marker associated with transcriptional termination, and possibly contributing to metacyclogenesis or vector-stage adaptation.

## Author summary

Chromatin is composed of a group of proteins called histones that are involved in compacting DNA and thereby affecting many DNA-associated mechanisms, such as replication and transcription. Histone variants share distinct primary sequences from the canonical ones and display different roles and genomic distributions. While variants of histone H3 and H2A are common in all eukaryotes, variants of H2B and H4 are unusual, except in trypanosomatids, a group of parasites that cause human and veterinary diseases. Among them, *Trypanosoma cruzi*, the causative agent of Chagas disease, exhibits variants for three core histones, but a variant of histone H4 (H4.V) had not been described. Here, we identify H4.V and demonstrate its preferential deposition at telomere-subtelomere interface, convergent strand-switch regions (cSSRs), and codirectional polycistronic transcription units (PTUs) interrupted by tDNA loci, likely marking transcription termination sites. Both histones, H4.V and H2B.V, are enriched in the nuclei of metacyclic trypomastigotes; however, H4.V is barely detectable in amastigotes and bloodstream trypomastigotes, while H2B.V is consistently present in all forms. Our findings reveal a novel histone variant with unique dynamics and epigenetic roles distinct from H2B.V in *T. cruzi*.

## Introduction

Chromatin comprises DNA and proteins that are organized into various structural levels, regulating genome access and, consequently, gene expression [1,2]. Histones constitute about 50% of chromatin mass and are classified as either canonical or variant histones. Canonical histones are typically abundant and expressed in the S-phase of the cell cycle; while histone variants differ from their canonical counterparts in their primary sequence, lower abundance levels and the ability to be expressed independently of the S-phase and in a tissue-specific manner [3–5]. Canonical and variant histones also differ in their gene and genome organization: while the former are intronless and organized in clusters, the latter are typically encoded by single-copy genes that contain introns [6].

Incorporation of histone variants into chromatin can alter the nucleosome-DNA interactions affecting chromatin organization and accessibility [5–7]. Histone variants have been identified across all studied model organisms — from yeast to plants and animals — and for all histone types, although rare examples exist for H2B and H4. Histone variants for histone H3 include the Centromere Protein A (CENPA) - located at centromeres [8] and H3.3 – associated with transcription activation [9]. MacroH2A and H2A.X are two histone H2A variants examples, associated with X chromosome inactivation [10] and DNA damage, respectively [11].

Limited H2B and H4 variants were identified in eukaryotes. Among the H2B variants, the H2BT was shown to be associated with spermatid differentiation in mammals [12] and H2B.1 specific to testis, oocytes, and zygotes [13]. In the parasite *Plasmodium falciparum* the histone H2B variant (H2B.Z) dimerizes with the H2A variant (H2A.Z) inhabiting the AT-rich promoter regions [14]. Regarding histone H4 variants (H4.V), fewer variants were identified, and their functional roles have been largely unexplored. H4 variants were found in *Gallus gallus* [15], in soybeans [16], and recently, a novel Hominidae-specific H4G was described which is predominantly localized in the nucleolus, influencing rRNA expression levels, protein synthesis, and cell cycle progression [17]. Examples of H2B.V and H4.V are also described in some trypanosomatids, as discussed below.

Trypanosomatids include some protozoa parasites that cause important medical and veterinary diseases. Among them, the causative agents of Chagas Disease (*Trypanosoma cruzi*), sleeping sickness (*T. brucei*), and leishmaniases (*Leishmania spp.*) are the most studied. These organisms exhibit particularities in their genome organization and gene regulation, such as the presence of polycistronic transcription units (PTUs), intronless genes, and trans-splicing [18]. The arrangement of PTUs in the genome gives rise to convergent strand switch regions (cSSRs), where the transcription of two PTUs ends, and divergent strand switch regions (dSSRs), where the transcription of two PTUs begins [18–20]. These regions encompass, respectively, the majority of transcription terminus site (TTS) and transcription start site (TSS) in trypanosomes, although head-to-tail PTU organization may also create TSSs and TTSs at non-SSR regions [21].

*Trypanosoma* chromatin is composed of histones that exhibit significant evolutionary divergence, notably in their N-terminal portion [22,23]. In contrast to other organisms, histone variants (H2A.Z, H2B.V, H3.V and H4.V) for all canonical histones were found in *T. brucei* [21]*.* Regions associated to transcription initiation and termination are demarcated by histone variants and some histone post-translational modifications (PTMs) across the *T. brucei* genome: H2A.Z, H2B.V, H4K10ac and H3K4me3 are located at TSSs [21,24–26]; while H3.V and H4.V, base J and H3K76me1 and H3K76me2 are enriched in TTSs [21,27–29]. H3.V, H4.V and base J are non-essential chromatin factors in *T. brucei*, but knockout parasites for all three factors exhibit critical changes in cell growth and replication [30]. H4.V in *T. brucei* was described to have 86% identity with the canonical H4 [21] and was shown to be the major determinant of transcription termination [30].

In the *T. cruzi* genome, all histone variants have been annotated except H4.V; however, only H2B.V has been previously characterized. We have shown that H2B.V localizes at dSSRs and certain tDNA loci. Parasites heterozygous knockout (HzKO) of H2B.V display increased differentiation into metacyclic forms and enhanced invasion of mammalian host cells [31]. Intriguingly, H2B.V is overexpressed in tissue-culture-derived trypomastigotes (TCTs) compared to epimastigotes and shows reduced chromatin affinity in TCTs [31]. However, it is not known whether *T. cruzi* encodes an H4.V and whether it has a preferential genomic distribution. Likewise, the expression dynamics and chromatin association of H2B.V and the potential H4 variant across the parasite’s life cycle and cell cycle are still unclear. This study addresses these gaps by identifying and characterizing a novel H4.V in *T. cruzi* that marks putative TTSs at cSSRs and at co-directional PTUs interrupted by some tDNA loci, and telomere-subtelomere interface. We also expand our investigation to better understand the expression patterns and chromatin interactions of both H4.V and H2B.V across life stages and throughout the cell cycle, revealing a variant-specific, dynamic regulatory landscape.

## Results

### *In Silico* Identification of a Novel *T. cruzi* H4.V

**Fig 1:**
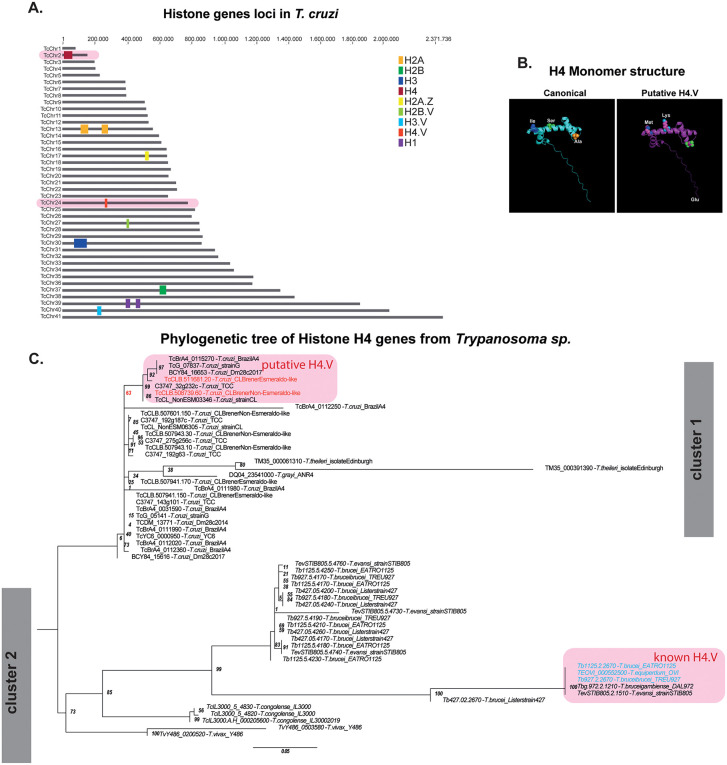
*In silico* identification of putative *T. cruzi* H4.V. **A.** canonical *T. cruzi* (CL Brener strain – S haplotype) histone loci are arranged *in tandem* in specific chromosomes, whereas variants histones are preferentially found in single copies and on different chromosomes. Chromosomes in red highlight canonical histone H4 and the putative H4.V locus. Notably, the histone H1 is the only histone that is not organized in tandem. **B.** Canonical histone H4 and putative H4.V protein structure prediction according to *AlphaFold*. Amino-acid differences between two histones are highlighted. **C.** Phylogenetic tree of the H4 histone gene family from the *Trypanosoma* genus. The tree was generated by nucleotide alignments and amino acid 3D structure of histone genes H4 and H4.V obtained from the TriTrypDB database and AlphaFold [80,81]. The species were grouped by the best probability of similarity from the ultrafast bootstrap values (1000 replicates) of the best maximum likelihood (best-fit model (TN + F + G4) - ML) shown at the tree nodes (confidence interval values). The CDSs represented in blue with a pink background are known as histone H4.V. The CDSs in red with a pink background are the putative H4.V from CL Brener strain investigated in this work. For building the tree file, identical H4 sequences of a given strain were considered only once (the identical IDs are listed in S1 Table). The tree layout was edited and exported from the FigTree software program.

To search for an H4.V in *T. cruzi* genome, we applied three *in silico* strategies, including analyses of genome locus, alignments, and phylogeny. Histone variant genes are generally dispersed throughout the genome, distinguishing them from the clustered arrangement of canonical histone genes [4,32,33]. This dispersed distribution reflects their specialized roles and unique regulatory mechanisms. We confirmed that in *T. cruzi* (CL Brener strain) single-copy of the variant histones H3.V, H2B.V, and H2A.Z are located on chromosomes 40, 27, and 17, respectively, while their canonical counterparts are arranged in tandem on different chromosomes (Fig 1A). For histone H4, we found that most *T. cruzi* H4 gene copies are located on TcChr2-S (11 copies) and arranged in tandem (Fig 1A), whereas a single-copy gene, TcCLB.511681.20, is located on TcChr24-S, suggesting it may encode a putative H4 variant. The CL Brener strain is a hybrid cell line that displays two haplotypes named Esmeraldo (S) and Non-Esmeraldo (P) like. We found a similar loci distribution of canonical H4 and its putative variant (TcCLB.508739.60) for the non-Esmeraldo like haplotype (8 copies of H4 at TcChr2-P and 1 copy of the putative H4.V at TcChr24-P).

Second, by aligning all the H4 sequences (12 sequences from S-haplotype and 9 from P- haplotype (P) (S1B Fig) we found that canonical histone H4 sequences from both haplotypes are conserved, differing in one amino acid at position 48 (V to I). Interestingly, the two putative H4.V single copy-genes shares 98% identity to the remaining histone H4 from CL Brener. While the putative H4.V from both haplotypes have a valine at position 48, they differ from the canonical H4 by three amino acids: I49M, which preserves the polarity of residues; S59K, which shifts from a polar (serine) to a cationic residue (lysine); and A100E, which shifts from a hydrophobic, nonpolar amino acid (alanine) to one with an anionic charge (glutamine). These amino acid substitutions are predicted to not induce major changes on the tertiary structure of the protein as inferred by AlphaFold predictions (Fig 1B).

Finally, from all histone H4 orthologs of the *Trypanosoma* genus (Figs 1C and S1A and S1 Table), we obtained a phylogenetic tree that evidenced two main clusters composed of histones H4 from *T. cruzi* (cluster 1) and other encompassing sequences from *T. brucei*, *T. congolense,* and *T. equiperdum* (cluster 2)*,* as expected by their phylogenetic differences*.* Within the second cluster, it is evidenced that known histone H4 variants (highlighted in blue) from *T. equiperdum* and *T. brucei* form a distinct cluster from their canonical counterparts. Within the cluster formed by *T. cruzi* histone H4, it is possible to observe that CL Brener strain sequences are dispersed into many subclusters, although one cluster diverged with 63 node ultrafast bootstrap (confidence interval – in red) from other H4 sequences of *T. cruzi*. Within this cluster, the putative H4.V are found. As a whole, TcCLB.508739.60 (P-haplotype) and TcCLB.511681.20 (S-haplotype) emerge as strong candidates for H4.V in *T. cruzi*.

### *T. cruzi* H4.V marks TTSs at cSSRs and co-directional PTUs interrupted by tDNAs, with additional enrichment at telomeric/subtelomeric regions

To explore the role of this putative histone variant, we used CRISPR-Cas9 to edit its locus obtaining ^Ty1-^H4.V epimastigote lineages (S2A Fig). The genome editing was confirmed through PCR (S2B Fig), Western Blotting (WB) of Whole Cell Extracts (WCE) from epimastigotes (S2C Fig). We showed that editing the H4.V locus did not affect parasite proliferation or differentiation (S2D and S2E Fig).

**Fig 2:**
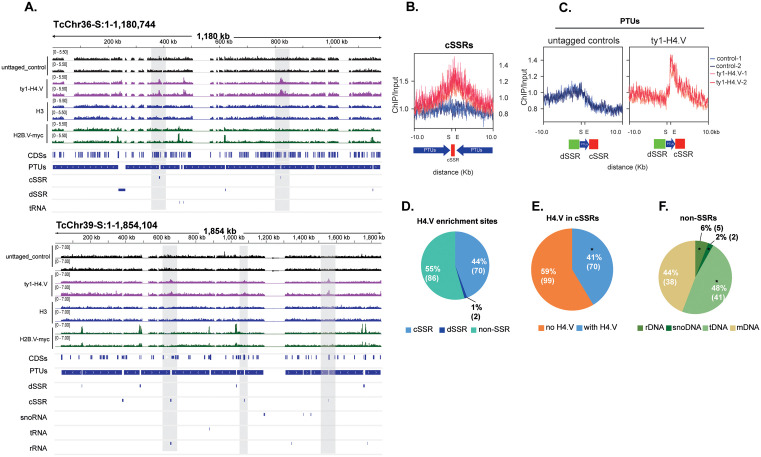
*T. cruzi* H4.V is enriched in cSSRs marking TTSs. **A.** IGV screenshot of H4.V ChIP-seq data from Chr36 and Chr39. The tracks “^ty1-^H4.V-epi”, “H2B.V-myc”, and “untagged control” represent the read coverage of the ratio values (ChIP/input) obtained by COVERnant (window size of 1001 bases per step of 201). The bed tracks represent cSSRs, dSSRs, snoRNAs, rRNAs and tDNAs loci. **B-C.** Plot profiles of histone H4.V enrichment detected by ChIP-seq assays in cSSRs (**B.**) and at polycistronic transcription units (**C.**). **D-F.** Pie charts representing the percentage of significant peaks identified by manual curation (Chi-squared test for given probabilities with simulated p-value (based on 1000 replicates) with calculation of the cut-off point for the standardized residues in genome; in cSSR (**E.**) cSSR p < 10^-15^; in non-SSRs (all genomic regions except SSRs) (**F.**) rDNA p < 10^-15^; snoDNA p < 10^-8^; tDNA p < 10^-15^. Non-SSRs represent 10.602 features; thus only 0.8% of them contains a H4.V enrichment peak. mDNA – loci coding for any mRNA. All summary plots were obtained by the COVERnant ratio values (ChIP/input), and all regions of interest (cSSR and PTUs) were fit into a scale-region represented as “Start” (S) and “End” (E).

Considering that *T. brucei* H4.V is deposited at TTSs [21], we performed ChIP-seq assays using parasites ^ty1-^H4.V and untagged parasites (Cas9 lineage) as a control. The reads were mapped using genome assemblies available at [34] and at [35] (obtained from long reads), obtaining 74.61% and 85.82% of mapped reads, respectively. We found that the putative H4.V is predominantly enriched in cSSRs (Figs 2A and S3), as illustrated by the summary plots of all cSSRs (Fig 2B) and polycistronic transcription units (Fig 2C). In the latter, H4.V enrichment is concentrated at the ends of polycistrons, where cSSRs are located. These results indicate that *T. cruzi* H4.V preferentially marks transcription termination regions, similarly to what has been observed for *T. brucei* H4.V [21], indicating a conserved feature among trypanosomes.

Enriched H4.V regions were compiled (S4A and S4B Fig and S3 Table), revealing that H4.V enrichment is distributed in cSSRs (44%), dSSRs (1%) and non-SSRs (55%) (Fig 2D). Non-SSRs (10.602 features) refers to all the remaining genomic features of the genome excluding SSRs. From the 169 cSSRs, 41% of them (n = 70) are enriched in H4.V (Fig 2E) (Chi-squared test, p-value <10^e-^^15^). Among H4.V enrichment in non-SSRs represented in Fig 2F, 48% represents loci close to tDNAs, 2% snDNAs and 6% rDNAs (Chi-squared test for tDNA p < 10^-15^, snoDNA p < 10^-8^, rDNA p < 10^-15^). Seventy-five percent of tDNAs (41/55) are in regions enriched in H4.V. The frequent localization of H4.V near loci transcribed by RNA polymerases other than RNA Pol II (which drives PTU transcription) suggests the establishment of new TTSs upstream of these regions as observed in *T. brucei* [21].

**Fig 3:**
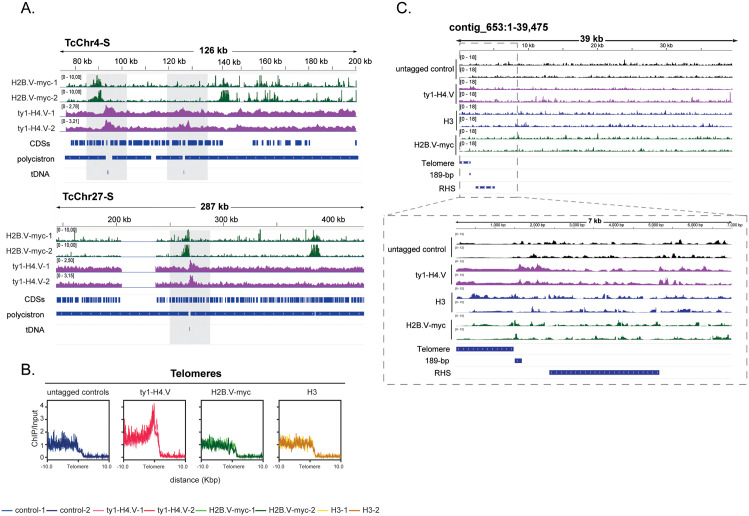
*T.* *cruzi* H4.V marks TTSs at co-directional PTUs interrupted by tDNAs, with additional enrichment at telomeric/subtelomeric regions. **A.** IGV snapshots of two chromosomes (Chr4-S and Chr27-S) harbouring codirectional PTUs exhibiting enrichment of either both or only one histone variant. Of note, co-directional PTUs were defined *in silico* according to the transcriptional orientation of their coding sequences and the presence of small non-coding RNA loci that interrupt a PTU. **B.** Plot profile of histone H4.V detected in subtelomeric and telomeric regions. All summary plots were obtained by the COVERnant ratio values (ChIP/input). Telomeric plot profiles were built considering telomeric repeats as a “reference-point” in deeptools using the 21 contigs containing the curated telomeric regions. **C.** IGV snapshot showing a 39-kb contig from the CL Brener long-read assembly, with a zoomed view highlighting H4.V enrichment at the interface between telomeric and subtelomeric regions. Additional examples are shown in S6 Fig.

*T. cruzi* tRNA genes are located alone or in clusters of 2–10 genes, within dSSRs, cSSRs or between codirectional PTUs. We previously found that H2B.V also localizes near tDNA loci [31] likely forming new TSSs in codirectional PTUs. Here, we found that 67% of tDNAs positioned between codirectional PTUs exhibit enrichment of both variants either marking new TSSs at their upstream (for H2B.V) and new TTSs at their downstream (for H4.V), Fig 3A.

H4.V is also enriched at the chromosome ends, likely corresponding to telomeric or subtelomeric regions (S5A Fig and S3 Table). Once these regions are not annotated in available databases, we improved the CL Brener genome assembly using long-read sequencing from [35], resulting on 21 putative telomeric regions (average and median size of 867 and 1024 bp, respectively). These regions were validated by the presence of the characteristic 189-bp junction sequence adjacent to telomeric repeats (S5 Table). This 189-bp sequence has been reported to flank all *T. cruzi* telomeres, marking their interface with subtelomeric regions [36].

Metaplot analyses revealed a clear enrichment of H4.V—but not H3 or H2B.V—near telomeric regions (Fig 3B). Among the 21 contigs containing the 189-bp junction adjacent to telomeric repeats, 95% showed H4.V enrichment extending across the 189-bp sequence and, on average, 1.8 kb into the adjacent subtelomeric region (Figs 3C, S5B and S6). Thus, H4.V accumulation is specifically detected at the interface between telomeric and subtelomeric regions.

### Dynamics of H2B.V and H4.V Levels Across the Cell Cycle

*T. cruzi* cell cycle phases display changes in morphology, transcription levels, proteomic and histone PTM patterns [37–40]. Using ^[3H]^lysine incorporation into synchronized parasites, it was shown that core histones, along with a subset of histone H1, are synthesized in coordination with DNA replication (S-phase) [41]. Histone variants can be expressed and deposited either in a replication-dependent or replication-independent manner [42,43]. To investigate the H2B.V and H4.V expression across the cell cycle, we performed immunofluorescence assays (IFA), and Image Flow Cytometry (IFC).

**Fig 4:**
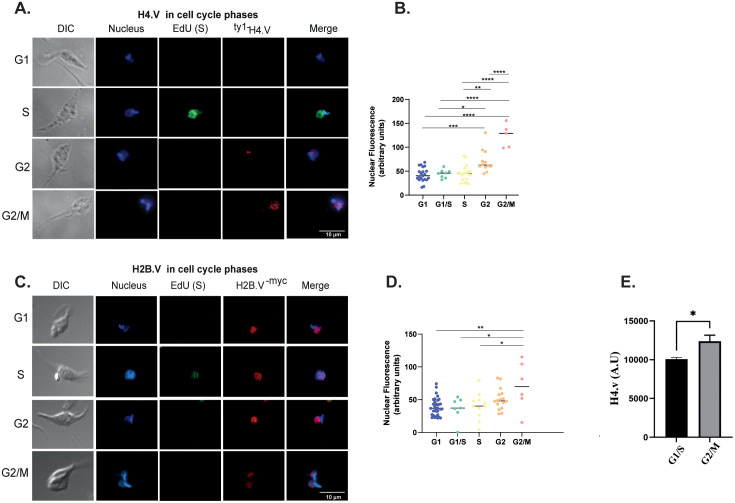
Nuclear distribution and abundance of histone variants in the *T. cruzi* cell cycle. IFA of ^ty1-^H4.V (**A.)** and H2B.V^-myc^ (**C.)** parasites from EdU incorporation assays in G1, S and G2/M cell cycle phases. Quantification of nuclear labelling from IFA of ^ty1-^H4.V **(B.)** and H2B.V^-myc^ **(D.)** parasites from two biological replicates. For H2B.V^-myc^ and ^ty1-^H4.V lines, 136 and 102 parasites were evaluated. Quantifications were performed in ImageJ considering a fixed nuclear area for all parasites. Ordinary one-way ANOVA with Tukey’s multiple comparisons test, where * (p < 0.05); and ** (p < 0.005). **F.** Barplots representing the median intensity values of ^ty1-^H4.V labelling in G1/S and G2/M from IFC data, normalized by nucleus area. Cell cycle determination was based on nuclear/kinetoplast areas obtained by DNA labelling (DRAQ5 detection) (S7 Fig). Upon filtering events, the estimated cell cycle gates were obtained (S7 B Fig) (see gating strategies at M&M section).

By IFA, H2B.V was consistently detected across all cell cycle phases, with its levels increasing as the cycle progresses (Fig 4A and 4B). Conversely, H4.V is slightly detected in G1 and S phases but their levels were more prominent in G2 and G2/M (Fig 4C and 4D). H4.V increased levels in later cell cycle phases were confirmed by IFC assays (Figs 4E and S7). Together, results revealed a progressive increase in both histone variants as cycle progresses.

### H2B.V and H4.V exhibit specific expression patterns across *T. cruzi* Life Stages

*T. cruzi* undergoes morphological and gene expression changes between replicative (epimastigotes and amastigotes) and non-replicative (trypomastigotes – TCTs and metacyclics) stages, adapting to distinct microenvironments [44–48]. Life form transition is also followed by alterations in nucleus and chromatin structure, including histone PTMs [49–52]. Given that the histone variants H2B.V and H4.V exhibit distinct characteristics mainly related to genomic distribution and cell cycle expression, we questioned whether their abundance might also vary within the nucleus during life cycle stages.

**Fig 5:**
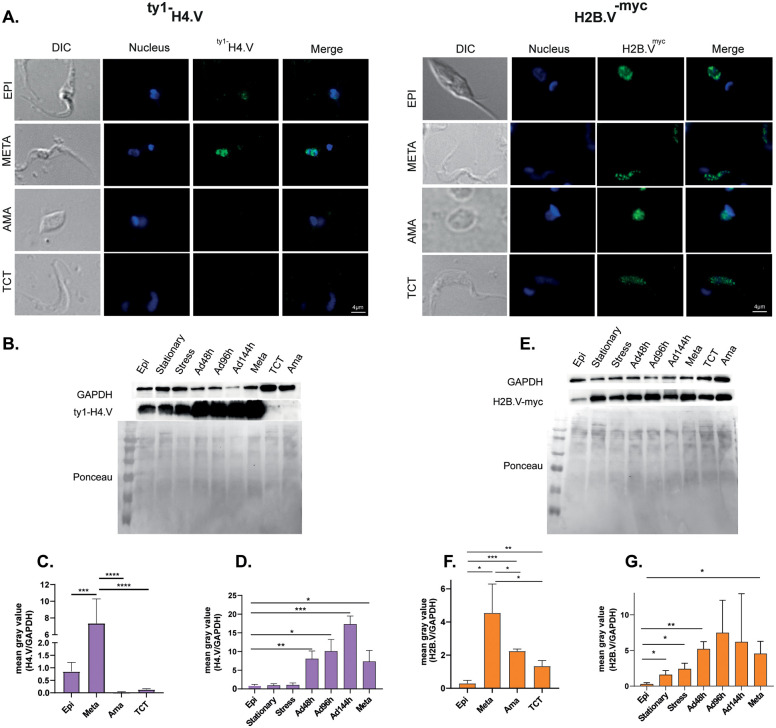
Subcellular localization and abundance of histone variants among *T. cruzi* life forms. **A.** Cellular localization of H2B.V and H4.V on different life stages of *T. cruzi*. Immunofluorescence of the parasites expressing either ^ty1-^H4.V (left) or H2B.V^-myc^ (right) in different life stages of *T. cruzi*. **B.** WB assays of WCE of parasites expressing ^ty1-^H4.V during metacyclogenesis and life forms. **C-D.** Quantification (mean grey value) of H4.V abundance normalized by GAPDH abundance obtained from (B.) showing significant differences of H2B.V among life forms (C) and metacyclogenesis **(D)**. **E.** WB assays of WCE of parasites expressing H2B.V^-myc^ during metacyclogenesis and life forms. **F-G.** Quantification (mean grey value) of H2B.V abundance normalized by GAPDH abundance obtained from (E.) showing significant differences of H2B.V among life forms (F) and metacyclogenesis **(G)**. Each well contains WCE of 5x10^6^ parasites. For all quantifications, we used an unpaired t-test of multiple comparisons, in which * = p < 0.05; ** = p < 0.01; *** = p < 0.005; and **** = p < 0.001. EPI = epimastigote; META = trypomastigote metacyclic; AMA = ama-like (extracellular amastigote); and TCT = tissue-culture-derived trypomastigote. WB were performed using 3 biological replicates.

H2B.V labelling was observed in the nuclei of all *T. cruzi* life forms by IFA (Fig 5A). This variant histone displays a punctate pattern distributed throughout the nucleus, excluding the nucleolar region. In the infective forms (TCT and metacyclic forms), the H2B.V staining resembles that in epimastigote forms but appears more dispersed, likely due to the elongated nuclear morphology of these forms. While H4.V showed more intense labelling in the metacyclic nuclei compared to other life forms. In epimastigotes, H4.V labelling varies in accordance with the cell cycle, as we have seen above. In contrast, H4.V is minimally detected in amastigotes and TCT forms in IFA assays. Unlike the punctate pattern observed for H2B.V, H4.V appears uniformly dispersed throughout the nucleus, with some metacyclic forms displaying a stronger signal.

By WB we detected that H2B.V abundance is more pronounced in TCT compared to epimastigote forms [31]. Here, we expand this analysis comparing the H2B.V abundance among all life forms (Fig 5B and 5C). Hence, H2B.V is more abundant in metacyclics, followed by amastigotes, TCTs and epimastigotes. Furthermore, changes in H2B.V abundance were detected along metacyclogenesis, mainly at initial stages of differentiation (epimastigote vs. stationary epimastigote vs. stress vs. Ad48h -p-value < 0.05) (Fig 5B and 5D).

In line with the predominant H4.V labelling in metacyclic forms observed by IFA (Fig 5A), WB assays confirmed a significantly higher H4.V abundance in these forms (Fig 5E and 5F). H4.V abundance shows a progressive increase throughout metacyclogenesis, as demonstrated in Fig 5G (unpaired multiple t-test, Epi stationary phase vs. ad144h, *P* < 0.0005). The presence of H4.V in epimastigotes, its increased expression in metacyclics, and its low expression levels in amastigotes and TCTs suggest that H4.V expression may be associated with the microenvironment of the invertebrate host likely affecting expression of surface virulence factors, as discussed below.

### Chromatin affinity of H2B.V, H4.V, and H3 are stronger in epimastigotes and metacyclics than in TCTs

**Fig 6:**
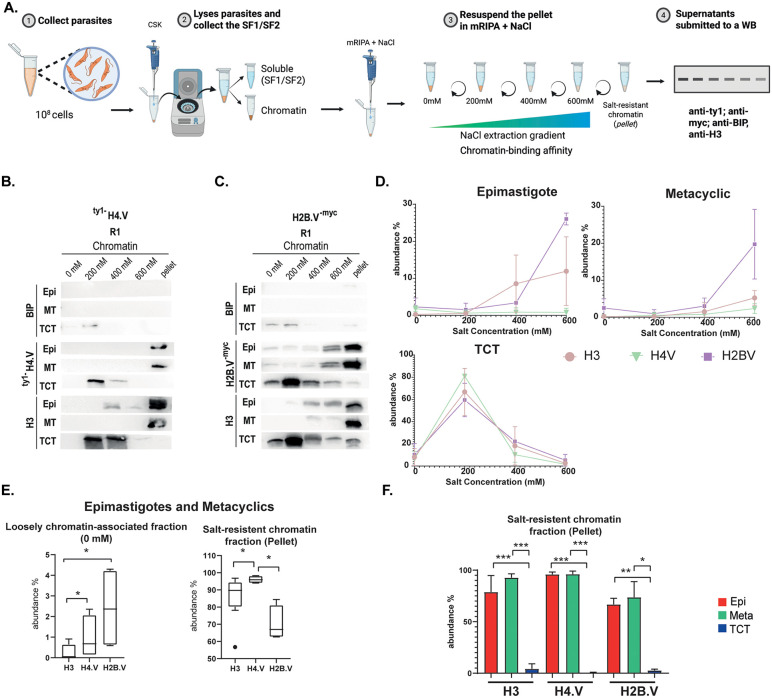
Assessment of the relative chromatin affinity of H2B.V, H4.V and canonical histone H3 in epimastigotes, metacyclics and TCTs life forms. **A.** Schematic representation of the salt extraction protocol for chromatin-associated proteins used to obtain the soluble fractions (shown in S8 Fig), the salt-extracted chromatin fractions, and the final salt-resistant chromatin (pellet). The workflow spans from parasite collection to Western blot (WB) analysis. Created with BioRender (Cunha, **J.**, 2025; https://BioRender.com/4z8rfby). Chromatin-soluble fractions from saline extraction (0, 200, 400 and 600 mM) of ^ty1-^H4.V (B.) and H2B.V^-myc^ (C.) parasites were obtained and evaluated by WB. WB were performed in biological replicates using antibodies against myc (H2B.V), ty1 (H4.V) and histone H3. Blots were imaged at different exposure times to ensure optimal signal detection. To account for differences in DNA content per in life form, volumes equivalent of 200 ng of DNA were applied for each life form, fractionated in 15% SDS-PAGE. **D.** Quantification (mean gray value) of the WB assays. To enable comparison across life forms and salt conditions, the sum of all mean gray value of each antibody signal (subtract by the background) in a given life form was normalized to 100%. **E.** Boxplots of relative abundance (in percentage) for the indicated histones at 0 mM salt extracts (left) and at pellet (right) for epimastigotes and metacyclics. Pairwise comparisons were additionally evaluated using the Mann–Whitney test where indicated. * p < 0.05. **F.** Bar plots showing the relative abundance (percentage) of the indicated histones across life forms. Statistical analysis was performed using ordinary one-way ANOVA followed by Tukey’s multiple comparisons test to compare each histone among life forms. *p < 0.05; ***p < 0.0001. Error bars represent SEM.

Histone variants and canonical histones can differently impact chromatin structure, either promoting a more accessible or stable chromatin state [53]. We showed that dSSRs and cSSRs have distinct chromatin landscape, mainly related to more open chromatin with lower nucleosome deposition at dSSRs compared to cSSRs [50,54]. Previously, we observed that H2B.V and H3 are released from chromatin at lower salt concentrations in TCTs compared to epimastigote forms, suggesting that these histones have a weaker binding affinity to TCTs’ chromatin [31]. In this study, we extend this analysis by investigating whether histone variants (H2B.V and H4.V) and a canonical histone (H3) display differential chromatin binding affinities across distinct life stages. To address this question, we obtained chromatin extracts and performed classical sequential chromatin salt-extraction assays (Fig 6A), which infer the strength of protein–DNA interactions based on protein solubility under increasing salt concentrations [55,56].

When comparing chromatin affinity across life forms, we observed that in epimastigotes and metacyclics, H2B.V (~3.3% and ~3%, respectively) begins dissociating from chromatin at 400 mM NaCl, retaining most of its interaction with chromatin (salt-resistant chromatin fraction – pellet) ~67% and ~73%, respectively). The canonical histone H3 (~8.5% and ~1.5%, respectively) follows a similar pattern, starting to dissociate at 400 mM NaCl, with a significant fraction remaining chromatin associated (~78% and ~93%, respectively). In contrast, H4.V remains bound at chromatin even when submitted to 600 mM NaCl (~96% for both) (Figs 6B, 6C and S8).

We performed linear regression analysis to infer differences into the dynamics of histone extraction in each life form, and although individual linear regressions suggested life-form dependent slopes for specific histones in epimastigotes and metacyclics, global ANCOVA revealed no significant differences among slopes (F(8,78)=1.226, p = 0.2953), while intercepts differed significantly (p = 0.0011), indicating variation in baseline abundance rather than differential salt responsiveness (Fig 6D). Then, we compared histone abundance in the loosely chromatin-associated fraction (0 mM NaCl) and observed that H2B.V levels were proportionally higher than those of canonical H3 (Fig 6E, p < 0.05). Conversely, H4.V was significantly enriched in the salt-resistant chromatin fraction (pellet) (Fig 6E, p < 0.05). Taken together, these data indicate that, in epimastigotes and metacyclics, H2B.V are more readily released under low-salt conditions compared to H3, consistent with comparatively weaker chromatin association. However, because regression slopes were not significantly different, the histones share comparable salt-dependent dissociation dynamics overall, and the observed differences are restricted to their relative distribution at the initial (0 mM) and final (salt-resistant) extraction steps.

In contrast, TCT’s chromatin exhibits markedly lower histone interaction affinity. All histones analysed dissociate at 200 mM NaCl (H3 ~ 62%, H4.V ~ 81%, and H2B.V ~ 60%), with a near-complete release occurring at 400 mM NaCl (Figs 6B-D and S8). Two-way ANOVA revealed a strong effect of salt concentration (F(4,25)=47.62, p < 0.0001), but no significant effect of histone identity (F(2,25)≈0, p > 0.9999) and no significant histone × salt interaction (F(8,25)=0.7213, p = 0.6713). Multiple comparisons detected no significant differences among histones at any salt concentration. These results indicate that, despite the non-linear extraction profile observed in TCT, the three histones display statistically indistinguishable extraction dynamics. Finally, comparison of chromatin pellet among histones and life forms, indicates statistical differences between TCTs versus epimastigotes and metacyclics for all three histones (One-way ANOVA; p adj < 0.001) (Fig 6F). This indicates that TCT’s chromatin is structurally distinct from that of the other compared life forms, particularly in terms of histone-binding affinity.

## Discussion

In this study, we identified a novel histone H4 variant and characterized its genomic distribution, abundance, and chromatin affinity across the *T. cruzi* life cycle and cell cycle. Evaluating also the variant H2B.V, we found that the two histone variants share both similar and different features related to their expression/abundance, genome distribution, and chromatin-binding affinities in *T. cruzi* life and cell cycle. The H4.V identification was supported by a combination of four main lines of evidence: (i) divergences in sequence and phylogeny compared to canonical H4; (ii) localization at unique genomic loci, distinct from the tandemly repeated canonical H4 sequences; (iii) stage- and cell cycle-specific abundance, suggesting specialized regulation; and (iv) specific genomic localization at cSSRs, near to some loci of tDNAs – likely demarcating TTSs, and at telomere-subtelomere interface. While evidence (iv) alone provides stronger support, it is the combination of all these features that firmly establishes H4.V as a histone variant. Notably, H4.V localization at cSSRs contrasts with the preferential association of H2B.V with dSSRs [21]. These conserved genomic distributions in trypanosomes highlight a potentially conserved role for histone variants in transcription initiation and termination regions in these organisms.

We previously showed that *T. cruzi* dSSRs have a more open chromatin state with lower nucleosome occupancy compared to cSSRs [54]. Here, we showed that, among the histones analyzed, a larger proportion of H2B.V was detected in the chromatin fraction released at 0 mM NaCl, indicating that this variant is more readily extractable under low-salt conditions compared to the canonical histone H3, supporting the presence of a more open chromatin structure at TSSs likely contributing to a configuration that is permissive to transcription initiation. In *T. brucei*, H2B.V–H2A.Z dimers are more unstable than canonical H2B–H2A dimers [21]. In contrast, H4.V was proportionally enriched in the salt-resistant chromatin fraction (pellet), suggesting comparatively stronger retention within chromatin. Although these observations do not directly demonstrate intrinsic differences in binding affinity, association with a more compact and stable chromatin state, or specific genomic localization, future studies should address these questions in greater detail, particularly given that, particularly given that,in eukaryotes, chromatin structure plays a role in slowing down transcription termination [57]. In trypanosomes, TTSs are enriched in base J, a modified thymine that is important for RNA polymerase II termination, as its absence leads to transcriptional readthrough [58]. Studies in *T. brucei* using parasites knockouts for all three TTS marks (H3.V, H4.V and base J) identified H4.V as a major signal for transcription termination [30]. Since all marks are deposited following DNA replication, the question remains as to which is deposited first and whether one influences the recruitment of the other — a topic that warrants further investigation. Considering that *T. cruzi* H4.V levels are controlled in a differentiation-specific manner, it should be determined whether H4.V is not as crucial for transcription termination in TCT and amastigote stages leading this function for other marks.

Aside from H4.V enrichment at cSSRs, we also found depositions of both H4.V and H2B.V in PTUs that are interrupted by loci of tRNAs, implying the creation of new TTSs and TSSs of RNA polymerase II into non-SSRs. This pattern was already observed in *T. brucei* [21], highlighting its conservation among trypanosomes. The H4.V enrichment in telomere-subtelomere interface is particularly noteworthy, as *T. cruzi* subtelomeric regions are known to house multigene families [59,60] associated with surface virulence factors (trans-sialidases) and chromatin structure regulation (RHS) [61] and the DGF-1 family. This pattern, together with the potential colocalization of H4.V and base J [21,29] in these regions, seems to be specific to *T. cruzi*, since in *T. brucei*, H4.V is absent from sub- and telomeric regions, where H3.V is instead enriched [21]. Notably, H3.V deletion in *T. brucei* disrupts VSG silencing [30], suggesting a role for histone variants in the regulation of subtelomeric gene expression. Altogether, these findings support the idea that histone variants H4.V and H3.V may have regulatory roles in the expression of virulence genes located in subtelomeric regions. Future studies using *T. cruzi* H4.V knockout lines will be essential to determine whether this variant directly contributes to the regulation of virulence factor gene expression. H4.V and H2B.V exhibit distinct abundance patterns throughout the parasite’s life cycle. Both variants are present in epimastigotes and metacyclic forms; however, H4.V is nearly absent in intracellular amastigotes and bloodstream trypomastigotes. This observation suggests a specialized role for H4.V in the insect vector environment or that H4.V expression is important for metacyclogenesis likely affecting surface virulence expression. Surface virulence transcripts, such as those encoding trans-sialidases and mucins, are more abundant in infective forms, particularly those living in the vertebrate host [62]. Considering that some of these genes are located in subtelomeric regions and that H4.V is associated with a compact chromatin state—which could hinder transcription in these areas—we hypothesize that the absence of H4.V in vertebrate stages (amastigotes and bloodstream trypomastigotes) may result in a more relaxed chromatin configuration at these loci, thereby facilitating the expression of virulence genes located in these regions.

During metacyclogenesis, H4.V levels progressively increase, whereas H2B.V undergoes a pronounced upregulation early in the differentiation process. Notably, the levels of both variants are higher in metacyclics compared to epimastigotes. Metacyclic forms are characterized by reduced transcriptional activity and more condensed chromatin dispersed throughout the parasite nucleus [51,52]. The enrichment of H4.V in chromosome ends, known to be enriched in heterochromatin [63], is consistent with the notion that this variant is more prevalent at salt-resistant chromatin fraction (pellet). Whether H4.V is directly involved with the generation of an heterochromatin state needs to be further evaluated, as well as the impact of H4.V higher enrichment in metacyclics. On the other hand, the enrichment of H2B.V in metacyclics appears counterintuitive, as nucleosomes containing H2B.V-H2A.Z dimers are typically associated with nucleosome destabilization and enhanced chromatin accessibility [21]. Further studies, such as ChIP-seq assays, are needed to confirm whether the genomic localization observed in epimastigotes is preserved in metacyclics or if these variants are redistributed more broadly across the genome, potentially leading to the increased global levels of H2B.V and H4.V in these forms.

Finally, our findings suggest potential implications for histone variants in the three-dimensional organization of chromatin. Recently, we found that cSSRs and tDNA loci of *T. cruzi* are preferentially located at the borders of chromatin-folding domains and evidenced that some tDNA loci interact with each other in a 3D organization [64]. Knockouts for H4.V and H3.V in *T. brucei* displayed critical changes in 3D organization [65]. Altogether, the deposition of *T. cruzi* histone variants at key genomic regions—such as dSSRs, cSSRs, tDNA loci, telomere-subtelomere interface—which occupy strategic positions in the 3D genome, suggests that these variants contribute to the establishment and/or maintenance of global chromatin architecture. Additionally, their varying abundance, along with their enrichment at TSSs and TTSs, suggest that transcription initiation and termination may also be modulated by the dynamic behaviour of these histone variants.

Overall, our study emphasizes the complex and dynamic roles of histone variants in *T. cruzi*, opening avenues for future research into their functional contributions to chromatin structure and transcription across the parasite’s life cycle. Comparison of H4.V and H2B.V features underscored the conserved role of histone variants in TSSs and TTSs in trypanosomes, while also highlighting their specific features in cell cycle and life forms.

## Materials and methods

### Phylogenetic analysis – Cladogram of Histone H4 Genes in *Trypanosoma spp*

H4 coding sequences (CDS) from the TriTrypDB database (https://tritrypdb.org/tritrypdb/app - downloaded at September 2022) were compiled into a FASTA file. Identical H4 sequences per strain were considered only once (S1 Table). Sequences exceeding 300 bp were eliminated from the dataset, realigned by MAFFT v7.475 program [66], and adjusted by manual curation with Aliview [67]. Translated sequences were aligned with predicted structural sequences from AlphaFold (“Q4CMF1.pdb” – putative H4.V and “Q4DDL6.pdb” – canonical H4) using PROMALSD3 [68]. Subsequently, the structural sequences obtained were excluded from the 3D alignment file and realigned with CDS nucleotides using the RevTrans 2.0 tool [69] to generate the final cladogram dataset. The maximum likelihood (ML) phylogenetic tree was estimated using the IQ-TREE program [70], applying the ultrafast bootstrap statistics algorithm with 1000 replicates to find the best-fit model. The found best-fit model was applied in a second phylogenetic tree performance. Tree files were analysed using the software FigTree v1.4.4 (http://tree.bio.ed.ac.uk/software/figtree/).

### Parasite cultures

*T. cruzi* CL Brener epimastigote forms were cultured in complete LIT medium [71] supplemented with 10% fetal bovine serum (FBS) and 0.5% hemin at 28°C. The constitutive Cas9 lineage [72], was supplemented with G418 (100 μg/mL); the ^ty1-^H4.V and H2B.V^-myc^ lineages with blasticidin (10 μg/mL) and puromycin (5 μg/mL), respectively. Metacyclic trypomastigotes were obtained following the protocol described by [73] with some alterations. The epimastigotes in stationary phase were incubated for 2 h in TAU medium(190 mM NaCl, 17 mM KCl, 2 mM CaCl_2_, 2 mM MgCl_2,_ 8 mM sodium phosphate buffer, pH 6.0) at 5 x 10^7^ parasites/mL, followed by dilution in TAU3AAG (TAU medium supplemented with 20 mM L-proline, 10 mM glucose, 50 mM glutamic acid, 2 mM aspartic acid) with 5 x 10^7^ parasites/mL. The cultures were then incubated horizontally at 28°C with 5% CO_2_ for 144 hours. Metacyclic purification was performed following the protocol described by [74]. *T. cruzi* trypomastigote forms (TCTs) were obtained from the supernatant of infected mammalian cells (LLCMK2) maintained in DMEM medium supplemented with 10% FBS at 37°C and 5% CO_2_ as described by [75]. After the 15th day of infection, extracellular amastigote forms were obtained and the released TCTs were incubated in the complete LIT medium for 24 hours to differentiate into amastigote-like forms [76]. Cells were counted using Neubauer chambers in biological triplicates.

### Growth curve

A total of 5 × 10^6^ epimastigotes/mL were transferred to a 24-well plate, and their growth was monitored over a period of 4 days. Parasite counts were conducted using a Neubauer chamber, and the means and standard deviations of the replicates were analysed relative to the growth of Cas9 lineage parasite (control).

### ^ty1-^H4.V parasite generation

^ty1-^H4.V lineage generation was obtained by CRISPR/Cas9 using lineages containing a T7 RNA polymerase and Cas9 integrated as described before [31,77] with some alterations. DNA from the plasmid pPOTv6-blast-mNG, containing the blasticidin resistance gene and the ty1 tag gene, along with the sgRNA targeting the 5’ UTR region of H4.V were amplified using Phusion High-Fidelity DNA Polymerase (ThermoFisher). The primers “Donor-H4V-Fw” and “Donor-H4V-Rv” were utilized to amplify the donor DNA from the plasmid pPOTv6 -blast-mNG. The PCR product comprised 30 base pairs of the homologous arm (the initial 30 nucleotides of the H4.V gene - (TcCLB.511681.20), excluding the methionine codon), three copies of the Ty1 tag sequence, the blasticidin resistance gene, and another 30 bp of homology arm (30 nucleotides from the intergenic region adjacent to the cleavage region). The donor DNA was inserted into the 5’ region of the H4.V locus for expression in the N-terminal portion of the H4.V protein. Primers “sgRNA-H4V-146” and “sg scaffold” were employed to amplify the sgDNA template. PCR products were purified using the QIAquick PCR Purification Kit and transfected into epimastigotes expressing Cas9 (CL Brener strain). Transfections were carried out using the same buffers described for the generation of H2B.V^-myc^ parasites [31], employing the Amaxa Nucleofactor electroporator (Lonza) with program X-014 in 0.2 cm cuvettes. The transfected parasites were then transferred to 5 mL of complete LIT medium and incubated at 28°C. After 24 hours, the cultures were supplemented with 10 μg/mL of blasticidin, and selected transfectants were maintained for two weeks. Parasite clones were obtained by limiting dilution with conditioned medium with 10 μg/mL blasticidin. Genome editing was confirmed by PCR using primers Fw-UP-blast-check [1], Fw-UP-^ty1-^check [2], H4V_Int3_Rv-check [3], and H4V_Rv-check [4], with primer sequences detailed in S2 Table.

### ChIP-seq ^ty1-^H4V

ChIP-seq experiments were performed following the protocol outlined in [31] with certain modifications. We used 3x10^8^ of epimastigote forms (^ty1-^H4.V and untagged Cas9 lineage parasites) in biological triplicates. For the sonication, the samples were resuspended in TELT buffer (50 mM Tris–HCl pH 8.0, 62.5 mM EDTA, 2.5 M LiCl, 4% Triton X-100) and sonicated in the Q800R3 sonicator (Qsonica) under the following conditions: 50% amplitude, 15 seconds on, 30 seconds off for 30 minutes at 4°C. The Dynabeads protein G (Thermo Fisher) ressuspended in blocking buffer (5 mg/mL of BSA diluted in PBS) containing 2.3 µg of anti-ty1 antibody (MA5–23513 - Invitrogen), was added to the supernatant and incubated at 4°C overnight with slow rotation. Following incubation, washes were performed with ice-cold RIPA buffer (50 mM HEPES pH 7.6, 1 mM EDTA, 0.7% sodium deoxycholate, 1% NP-40, 0.5 M LiCl, and 1 mM PMSF), followed by a final wash with TE buffer (10 mM Tris-HCl pH 8.0 and 1 mM EDTA pH 8.0). The chromatin was eluted in elution buffer (50 mM Tris-HCl pH 8.0, 10 mM EDTA pH 8.0, and 1% SDS) at 65°C for 30 minutes. After DNA purification, described in [31], ChIP and input samples were used to construct Illumina DNA Prep (M) Tagmentation libraries using indexes adapters (IDT for Illumina DNA/RNA) and subsequently sequenced using Illumina NextSeq 1000/2000 (100 bp - pair end).

### ChIP-seq data analysis

ChIP-seq processing and analysis followed the protocol outlined in [31] with slight modifications. Reads were filtered per quality using Trimmomatic version 0.39 [78], trimming 11 nucleotides from the 5’ ends, utilizing a read size of 50N to minimize errors, employing a moderate penalty stiffness (0.05), trimmed at the 3’ end starting at nucleotide 89, and those shorter than 36 nucleotides were discarded. Quality control was conducted using FastQC version 0.11.8 [79]. H4.V ChIP-seq were aligned to the CL Brener Esmeraldo-like strain (version 32 – TriTrypDB) using Bowtie2 version 2.3.5.1 [80] with stringent parameters (very-sensitive-local: -D 25 -R 4 -N 1 -L 19 -i S, 1,0.40 --nceil L,0,0.15). Coverage data were generated using COVERnant v.0.3.2 (https://github.com/konrad/COVERnant), resulting in ratio values derived from paired analyses of each ChIP sample versus its corresponding input. The output was saved as.wig files and visualized using IGV (http://software.broadinstitute.org/software/igv/). To identify genomic regions enriched in H4.V, coverage data were manually curated by comparing regions of enrichment in the Ty1-H4.V ChIP/input samples with those from the untagged ChIP/input control. The H4.V enriched genomic coordinates were listed in S3 Table. The ChIP-seq coverage data was analysed using a custom GFF file (from version 32), annotated using a script available at (https://github.com/alexranieri/annotatePolycistron), which annotates polycistrons, dSSRs, and cSSRs (coordinates are shown at S4 Table). In brief, the orientation of all coding DNA sequences (CDSs) was used to define PTU coordinates by grouping adjacent CDSs transcribed in the same direction into a single PTU. A PTU ends at the last CDS before an adjacent CDS transcribed in the opposite direction or when encountering any non-protein-coding gene (e.g., tRNA, snoRNA, snRNA, or rRNA). In such cases, we defined the regions as *putative co-directional PTUs*. Since no TSS mapping has been performed in *T. cruzi*, the only *putative* co-directional PTUs that can be identified are those where a PTU is interrupted by a small non-coding RNA transcribed by a polymerase other than RNAPII. Summary plot and heatmap graphs were generated using the computeMatrix (with scale-regions and skipZeros options) and plotHeatmap (with hierarchical clustering) functions from deepTools2 [81].

### Immunofluorescence Assays (IFA) and Click-iT Reaction

For IFA, the parasites were adhered to poly-lysine-treated 8-well slides and fixed with 4% ultrapure paraformaldehyde. After fixation, slides were incubated with PBS supplemented with 20 mM glycine, permeabilized with 0.2% NP40 in PBS, blocked with 50% SFB in PBS and 3% BSA for 1 hour, then incubated with 6 M guanidine chloride in PBS, and washed. Slides were incubated for 1 hour with anti-Myc (Cell Signaling, 1:2000) and anti-ty1 (Thermo Fisher Scientific, 1:200), followed by anti-mouse Alexa Fluor 488 (Thermo Fisher Scientific, 1:2000). Parasites expressing the Cas9 protein (Cas9 lineage) served as the control group. For S-phase detection, epimastigotes were previously incubated in complete LIT medium supplemented with 10 mM EdU for 20 minutes, as described by [37], subsequently the cells were submitted to IFA with myc or ty1 and Alexa 555 anti-mouse secondary antibodies (Thermo Fisher Scientific), and then followed for Click-iT EdU Imaging Kit with Alexa Fluor 488 (Thermo Fisher Scientific) to detect Edu-labelled DNA. All slides were mounted with Vectashield plus DAPI. Slides were viewed at 100X magnification on an inverted OLYMPUS model IX81 microscope. The cell cycle phase in IFA was determined by morphological markers based on the number of flagella, kinetoplasts, nuclei, and EdU incorporation, as described previously [37].

### Imaging Flow Cytometry (IFC)

Imaging Flow Cytometry (IFC). For assays, a total of 2x10^7^ cells in the exponential growth phase were washed with PBS and centrifuged at 1500g for 10 minutes. Subsequently, fixation was carried out using 4% paraformaldehyde (PFA) for 3 minutes, followed by washing with PBS 1x. (Note: all washes were performed by centrifugation at 1500g for 3 minutes). After fixation, cells were permeabilized with 0.1% PBS/tween-20 for 10 minutes and blocked with 3% PBS/BSA for 1 hour. Incubation with the primary antibody (anti-H3 1:2000 and anti-ty1 1:50, respectively) was conducted according to the manufacturer’s recommendations, diluted in PBS/BSA 2% for 1 hour and overnight, respectively. Following incubation with the primary antibody, three washes were performed, followed by incubation with the secondary antibody (anti-mouse PE - Texas Red 1:5000 and anti-rabbit Alexa Flour 488). Then, an incubation with the DNA marker Draq5 (1:500), diluted in PBS/ BSA 2% was performed for 1h30. Finally, the cells were washed three times and stored at 4°C for subsequent analysis on the Amnis cytometer.

### IFC data analysis

Data acquisition was conducted with 20,000 events by multispectral Image Flow Cytometry (ImageStreamx mark II Imaging flow-cytometer: Amnis Corp, Seattle, WA, Part of Luminex) using a 60 × objective. The selection/gating strategy involved the exclusion of aggregates (area x aspect ratio features, spot cutting), followed by the selection of focused cells based on the Gradient RMs feature. Subsequently, three steps were applied: 1. Selection of H3-positive cells based on “Max Pixel Intensity” versus “Intensity” of H3 (Alexa Fluor 488 in channel 02); 2. From the population selected in step 1, cells in G1/S and G2/M were discriminated based on nuclear “Area Intensity” versus “Width Intensity,” combined with the kinetoplast signal (DRAQ5, channel 05). Using the imaging capabilities of ImageStream, gating was refined as follows: cells with small nuclear area, a single kinetoplast, and one flagellum were classified as G1/S; cells with enlarged nuclear area, two kinetoplasts, and two flagella were classified as G2/M.3. From cells obtained in 2, evaluation of the percentage of ^ty1-^H4.V-positive cells and their intensity levels (median) in the G1/S and G2/M populations derived from the H3-positive cells. Data were analyzed using Idea 6.3 analysis software (Amnis Corp.) and are presented as mean ± standard deviation. Student´s t test, * p < 0,01.

### Salt extraction of chromatin-associated proteins

The salt extraction procedure was performed as described in [31] and based on [82]. Cytoplasmic and nuclear membranes of approximately 10⁸ parasites (epimastigotes, metacyclics, and TCTs) expressing H2B.V-myc and Ty1-H4.V were lysed using 100 μl of CSK buffer (100 mM NaCl, 10 mM Tris-HCl pH 7.4, 3 mM MgCl₂, 300 mM sucrose, 0.1% Triton X-100) supplemented with protease inhibitors (Sigma), as previously described [83]. This procedure generated soluble cytoplasmic and nuclear fractions (S1 and S2 Figs). The remaining pellet, mainly composed of chromatin, was subjected to sequential salt extraction using 100 μl of modified RIPA buffer containing 0, 200, 400, and 600 mM NaCl. The final pellet was resuspended in 100 μl of 1 × SDS-sample buffer. For each life form, equal volumes of salt extracts and the final pellet were analyzed by SDS–PAGE (15%). To account for the different DNA content of each life form, DNA concentration was quantified using a Qubit High Sensitivity kit (Thermo Fisher Scientific), and equivalent amounts corresponding to 200 ng of DNA were loaded for each sample.

### Western Blot (WB)

WB assays were conducted using samples from whole cell extracts (WCE) and salt gradient extractions. Membranes were blocked in a 5% solution of skimmed milk powder in TBS-T for 1 hour, followed by overnight incubation with primary antibodies at the following dilutions: anti-myc (Cell Signalling) 1:8000, anti-H3 (Abcam) 1:8000, anti-BIP 1:1000, anti-GAPDH 1:2000 [84], and anti-ty1 (Thermo - MA5–23513) 1:500. Secondary antibodies conjugated to peroxidase (Thermo) were used at 1:3000. All washes were performed by shaking three times for 6 minutes. Membranes were visualized using ECL Western Blotting Substrate in the chemiluminescence light in Uvitec 4.7 Cambridge document photo system. Quantifications of WB results were obtained from the mean gray intensity (ImageJ). Normalizations were performed by the mean gray intensity of GAPDH labelling.

### Obtaining Telomeric and Subtelomeric Interface from an improved CL Brener genome assembly

The CL Brener genome sequence was obtained through long-read sequencing, sequenced using MinION technology (Oxford Nanopore Technologies) [35]. Fastq files underwent filtration using nanofilt to retain reads with a minimum quality Q score of 15 and a minimum size of 10 kb. Filtering yielded 529,882 reads with an N50 estimated at 33,447 bp. This read set was utilized to assemble a draft genome using Flye version 2.9.1-b1780 [85] with default parameters, resulting in a genome comprising 334 contigs with an average depth of 204 X, totalling 60.5 Mb and an approximate N50 of 282 kb. The genome underwent correction with two successive rounds using Medaka software (polish), culminating in the generation of a consensus genome. Telomeric regions were annotated by performing BLAST searches on contigs containing the telomeric sequence TTAGGGn (3x) of approximately 1 Kb, including the complementary reverse, against the consensus genome, yielding only one match per contig. Consequently, repeats were identified in 41 contigs. Furthermore, the Geneious Prime program (available at http://www.geneious.com/) was employed, utilizing the “similarity annotation” tool to search for TTAGGGn(3x) in the 41 detected contigs. Telomeric regions were further filtered to retain only those located at the distal ends of the scaffolds (either at the first or last nucleotide position), resulting in a final set of 25 regions. Four contigs were excluded due to the presence of gaps between two telomeric sequences. The 189-bp junction sequence adjacent to the telomeres [36] was annotated using BLAST searches, considering hits with a minimum of 80% sequence identity. All 21 validated telomeric regions were found to be immediately followed by this junction sequence, further confirming their telomeric identity (S5 Table). It is noteworthy that the annotation lacks exact coordinates but represents the closest match respecting the repetition limit (TTAGGG).

## Supporting information

S1 Fig Identification *of T. cruzi* putative H4.V. A. H4.V identification by phylogenic analysis. Triangle graph of the phylogenetic signal of maximum-likelihood probability calculation showing the percentages of the three topologies represented in the left triangle, referring to the tree in Fig 1A. The triangle on the right represents the attractor that, by similarity, distributes the mapped gene sequences. It was observed that the sum of the percentage of attractor vertices in the triangle on the right is greater than 60%, so the best topological model has a good phylogenetic signal, which is why it was used in the generated phylogenetic tree (ultrafast bootstrap values by 1000 replicates - best-fit model: TN + F + G4). B. Canonical histone H4 and putative histone H4.V from *T. cruzi* (CL Brener strain – S- and P- haplotype). Nucleotide and amino acid alignments were performed at Clustal Omega Clustal Omega - Multiple Sequence Alignment from EMBL-EBI plotted on Geneious Prime software. Nucleotides and amino acids highlighted in colour indicate mismatches in the alignment.
(PDF)

S2 Fig ^ty1-^H4.V parasites generated by CRISPR-Cas9. A.Scheme representing the H4.V locus before (top) and after (bottom) gene editing by CRISPR. Alignment of the upstream coding regions of the H4.V and H4 loci, showing the primer (H4V_Nterminus_146) used to generate the sgRNA.The donor DNA has three copies of ty-1 sequences; the 5’ UTR of *T. brucei* actin, 3’UTR of aldolase and the 5’ UTR of aldolase (dark grey); the blasticidin resistance gene (red); and homology arm (Harm-dark blue) that are homologous to the 5’ UTR of H4.V. The red arrows indicate the primers that were used to validate the genome edition, and black arrows indicate the gene transcriptional direction. IGR - IGR represents the intergenic region, and H-Arm represents the homologous arm. **B**. PCRs of ^ty1-^H4.V parasites using the indicate pair of primers. Amplicons were fractionated by 1% agarose gel showing the insertion of the t ty1 sequences specifically at the H4.V locus. Primers were designed to anneal specifically to the blasticidin-resistance gene (Primer 1), the ty1 sequence (Primer 2), and the H4.V sequence (Primers 3, 4, and 5).Primer 5 was designed to anneal specifically to the H4.V sequence, which differs from canonical H4 by three nucleotides, including at the most distal 3′ end. The two terminal 3′ nucleotides of Primer 3 are unique to H4.V, ensuring specific amplification compared to canonical H4.The genomic DNA of parasites expressing Cas9 were used as negative control (C-). Results do not allow us to infer whether one or both alleles were edited. **C.** Western blot assay using anti-ty1 confirmed the H4.V expression in epimastigotes clones. Non-transfected parasites were used as negative control (Ctl). **D.** Parasites expressing Cas9 and ^ty1-^H4.V growth curves (log10). Unpaired T-test of area under the curve, p = ns. **E.** Metacyclic trypomastigotes expressing Cas9 or ^Ty1-^H4.V were quantified in RPMI culture supernatants after 8 days. Unpaired T-test, p = ns (p = 0.1609). The experiments were performed in biological triplicates. ns: no statistically significant differences.
(PDF)

S3 Fig Distribution and validation of H4.V enrichment. A. Plot Enrichment graph (from deepTools) from all H4.V manually enriched genomic regions confirms higher enrichment of H4.V in all ^Ty1-^H4.V ChIP sample replicates compared to control samples (input control-Cas9, ChIP control-Cas9, input ^Ty1-^H4.V). B. Quantification between enriched genomic coordinates from ChIP and input samples described in (C.). Multiple comparisons One-way ANOVA were performed in which * (p < 0.05) and ** (p < 0.005).
(PDF)

S4 Fig IGV snapshots of H4.V and H2B.V enriched in three chromosomes. The tracks “^ty1-^H4.V-epi”, “H2B.V-myc”, and “untagged control” represent the read coverage of the ratio values (ChIP/input) obtained by COVERnant (window size of 1001 bases per step of 201). The bed tracks represent cSSRs, dSSRs, snoRNAs, rRNAs and tDNAs loci.
(PDF)

S5 Fig H4.V enrichment in chromosome ends. A. IGV screenshot of ^Ty1-^H4.V ChIP-seq data from the chromosome 23S. The track “ty1-H4.V-epi”, “untagged control”, “H2B.V-myc” and “H3” represent *.wig files from the ratio values (ChIP/input) obtained by COVERnant (window of 1001 bases per step of 201). The bed tracks in green represent the conserved genome compartment, in red the disruptive compartment, and in yellow genes RHS. Dashed box represent enrichment of histone H4.V at chromosome ends. B. Plot profile of histone H4.V, H2B.V and H3 (from ChIP-seq data) detected in the 189 bp sequence present in the transition of all sub- and telomeric regions of *T. cruzi*. All summary plots were obtained by the COVERnant ratio values (ChIP/input). Plot profiles were built considering the 189 bp sequence as a “reference-point” in deeptools using the 21 contigs containing the curated telomeric regions.
(PDF)

S6 Fig IGV snapshots of H4.V, H2B.V and H3 enrichement in two CL Brener contigs from long reads. The telomeric and subtelomeric regions were highlighted. The bed tracks represent the telomeric repeats, the 189-bp and when annotated, the trans-sialidases, RHSs, mucins and DGF-1.
(PDF)

S7 Fig IFC analysis of ^ty1-^H4.V parasites. A. Fluorescence intensity plots showing the following labels: Alexa Fluor 488 (histone H3) in channel 02 (ch02); DRAQ5 (nucleus) in channel 05 (ch05), PE-Texas Red (histone H4.V) in channel 04 (ch04). B. Sequential gating strategy to extract H4.V abundance on different cell cycle phases. Initial gate selection of histone H3-positive cells based on “Max Pixel Intensity” versus “Intensity of H3” (top left); followed by cell cycle phase discrimination (G1/S and G2/M) based on nuclear Area Intensity versus Width Intensity (DRAQ5 detection) combined with kinetoplast/flagella quantification (top right); subsequent selection of ^ty1-^H4.V-positive cells (“Max Pixel Intensity” versus “Intensity”) within the H3-positive populations in each cell cycle phase (bottom quadrants). C. Selected images of triple-stained epimastigotes labelled for histone H3, histone ^ty1-^H4.V, and nuclear staining, with corresponding merged images across cell cycle phases obtained from IFC. D. Graph illustrating the percentage of positive cells for both H3 and H4.V in each cell cycle phase. Graphs and images were generated using the Idea 6.3 analysis software (Amnis Corp.).
(PDF)

S8 Fig Salt-extracted chromatin is devoid of the cytosolic mark - BiP. A. S1 and S2 Figs, soluble fractions from both cytosol and nucleus, from parasites ^ty1-^H4.V and H2B.V^-myc^ were fractionated by SDS-PAGE and evaluated by WB using antibodies against BiP. B. WB analysis analysis of an independent second biological replicate corresponding to the experiment shown in Fig 6. C. Quantification of WB signal intensity (mean gray value with background subtraction) combining both biological replicates. From left to right: H4.V, H2B.V and H3. To enable comparison across life forms and salt conditions, the sum of all mean gray value of each antibody signal in a given life form was normalized to 100%.
(PDF)

S1 Table IDs and CDS from histone H4 of Trypanosoma sp. (XLSX)

S2 Table Primers. (XLSX)

S3 Table H4.V peaks in the CL Brener Esmeraldo-like haplotype.
(XLSX)

S4 Table CL Brener Esmeraldo-like haplotype all features coordinates. (XLSX)

S5 Table Subtelomeric and Telomeric coordinates from CL Brener assembly obtained from long-reads. (XLSX)
