# Supplementary figures and images for: New Histone H4 variant and H2B variant Exhibits distinct genomic distributions, chromatin affinities, and dynamics throughout life and cell cycle of Trypanosoma cruzi
Source: PLoS Pathog. 2026 Mar 31;22(3):e1013310. doi: 10.1371/journal.ppat.1013310 (PMC13052989; doi:10.1371/journal.ppat.1013310)

## SF1

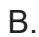

## Canonical

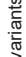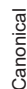

variants

Supplement: S1 Fig — H4.V identification by phylogenic analysis. Triangle graph of the phylogenetic signal of maximum-likelihood probability calculation showing the percentages of the three topologies represented in the left triangle, referring to the tree in Fig 1A. The triangle on the right represents the attractor that, by similarity, distributes the mapped gene sequences. It was observed that the sum of the percentage of attractor vertices in the triangle on the right is greater than 60%, so the best topological model has a good phylogenetic signal, which is why it was used in the generated phylogenetic tree (ultrafast bootstrap values by 1000 replicates - best-fit model: TN + F + G4). B. Canonical histone H4 and putative histone H4.V from T. cruzi (CL Brener strain – S- and P- haplotype). Nucleotide and amino acid alignments were performed at Clustal Omega Clustal Omega - Multiple Sequence Alignment from EMBL-EBI plotted on Geneious Prime software. Nucleotides and amino acids highlighted in colour indicate mismatches in the alignment. (PDF) [file ppat.1013310.s001.pdf]

A.

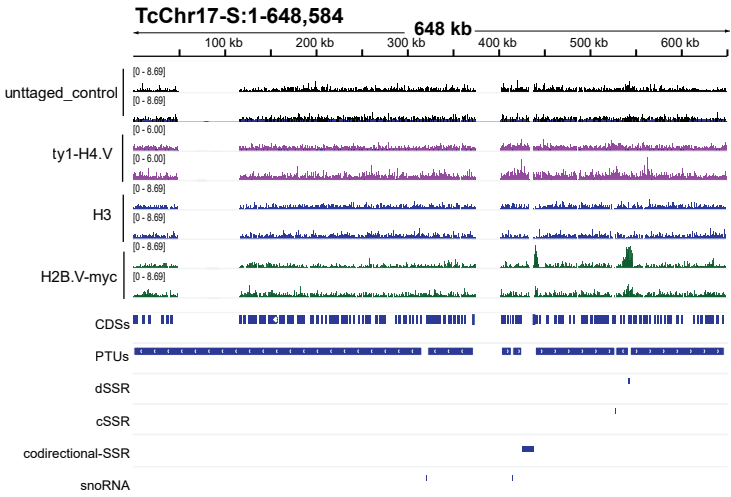

B.

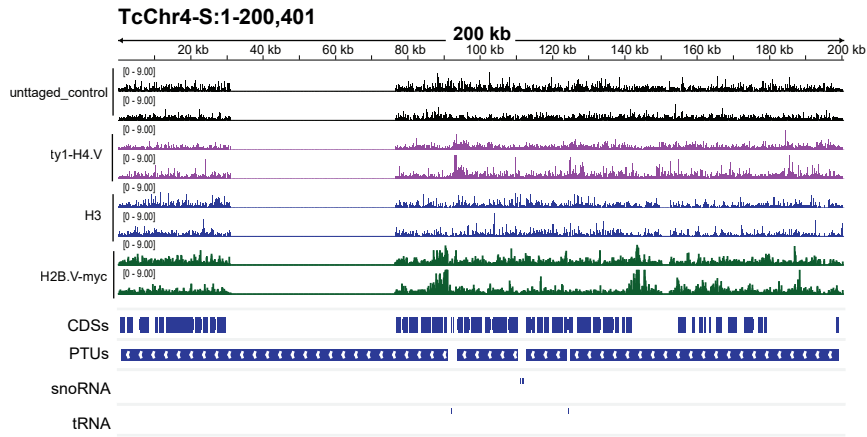

C.

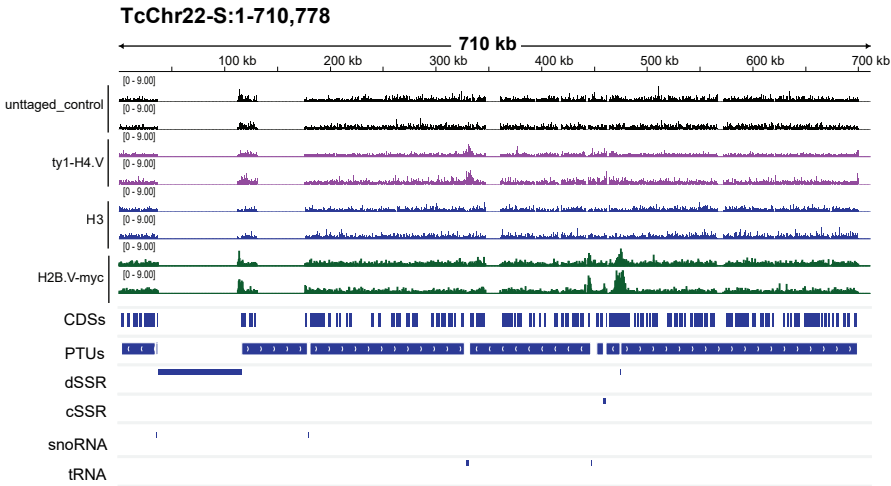

Supplement: S3 Fig — A. Plot Enrichment graph (from deepTools) from all H4.V manually enriched genomic regions confirms higher enrichment of H4.V in all Ty1-H4.V ChIP sample replicates compared to control samples (input control-Cas9, ChIP control-Cas9, input Ty1-H4.V). B. Quantification between enriched genomic coordinates from ChIP and input samples described in (C.). Multiple comparisons One-way ANOVA were performed in which * (p < 0.05) and ** (p < 0.005). (PDF) [file ppat.1013310.s003.pdf]

A.

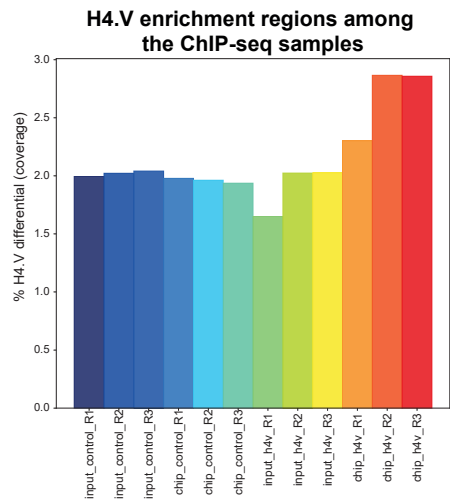

B.

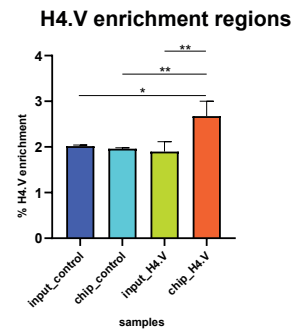

Supplement: S4 Fig — The tracks “ty1-H4.V-epi”, “H2B.V-myc”, and “untagged control” represent the read coverage of the ratio values (ChIP/input) obtained by COVERnant (window size of 1001 bases per step of 201). The bed tracks represent cSSRs, dSSRs, snoRNAs, rRNAs and tDNAs loci. (PDF) [file ppat.1013310.s004.pdf]

A. End of Chromosome 23-S

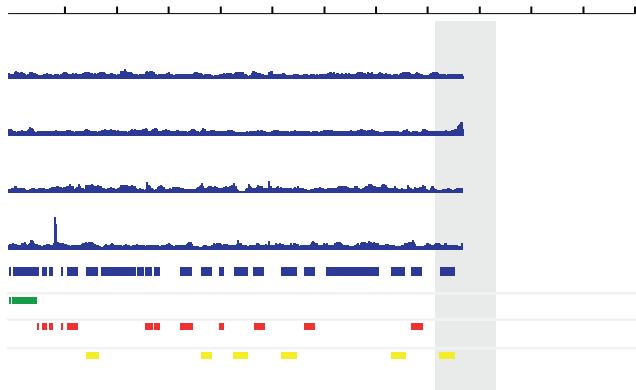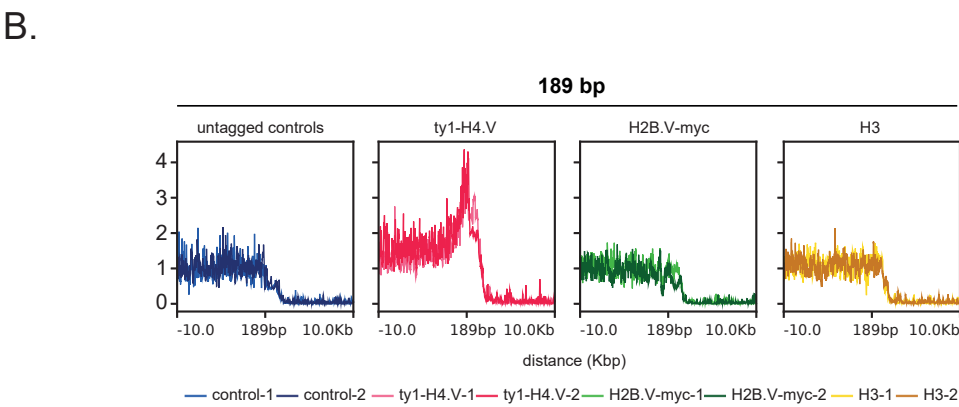

Supplement: S5 Fig — A. IGV screenshot of Ty1-H4.V ChIP-seq data from the chromosome 23S. The track “ty1-H4.V-epi”, “untagged control”, “H2B.V-myc” and “H3” represent *.wig files from the ratio values (ChIP/input) obtained by COVERnant (window of 1001 bases per step of 201). The bed tracks in green represent the conserved genome compartment, in red the disruptive compartment, and in yellow genes RHS. Dashed box represent enrichment of histone H4.V at chromosome ends. B. Plot profile of histone H4.V, H2B.V and H3 (from ChIP-seq data) detected in the 189 bp sequence present in the transition of all sub- and telomeric regions of T. cruzi. All summary plots were obtained by the COVERnant ratio values (ChIP/input). Plot profiles were built considering the 189 bp sequence as a “reference-point” in deeptools using the 21 contigs containing the curated telomeric regions. (PDF) [file ppat.1013310.s005.pdf]

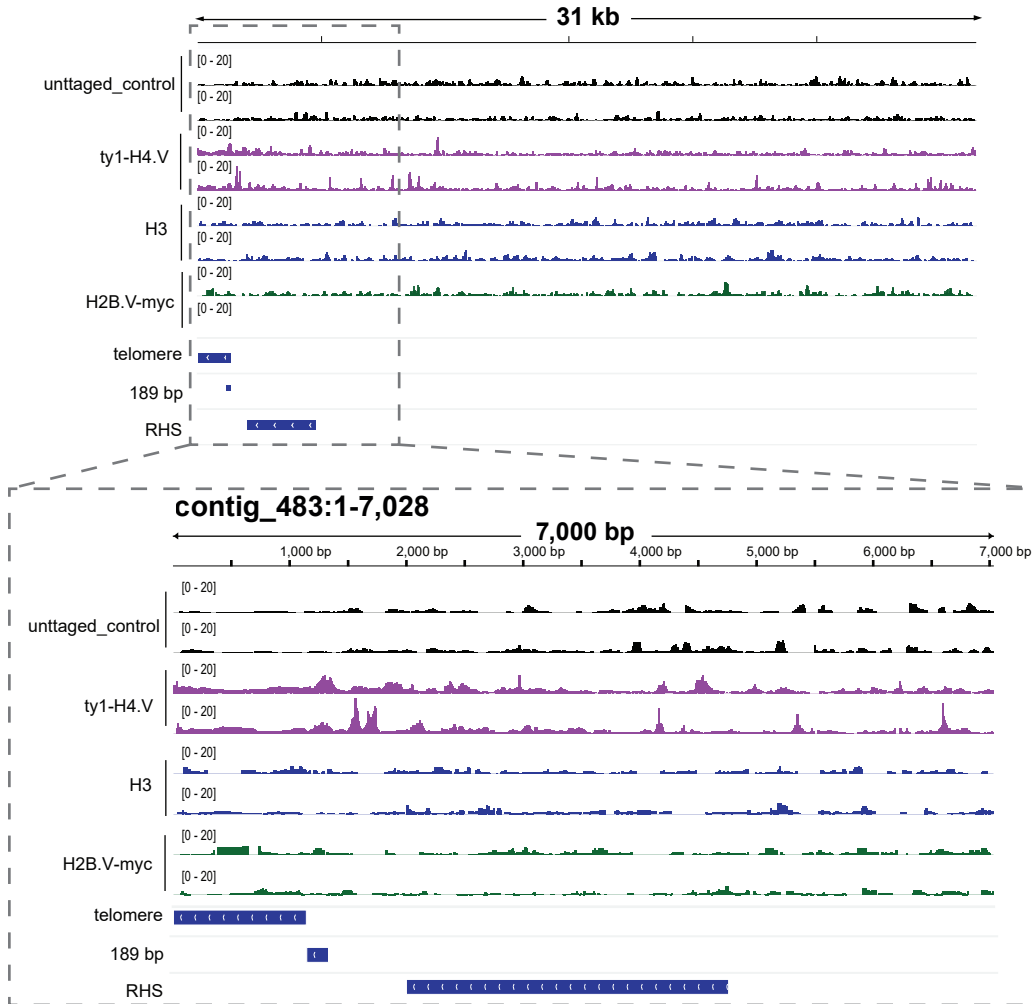

contig\_764:1-151,480

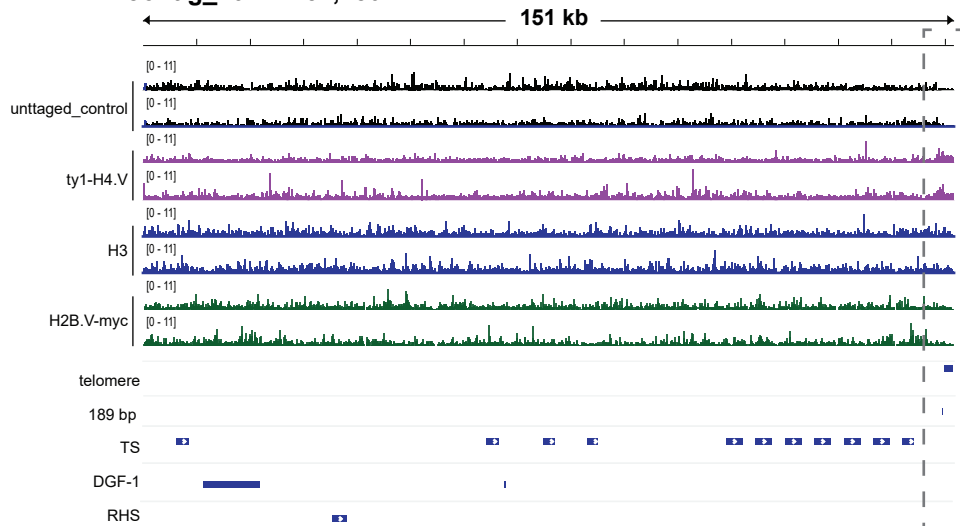

contig\_764:144,467-151,480

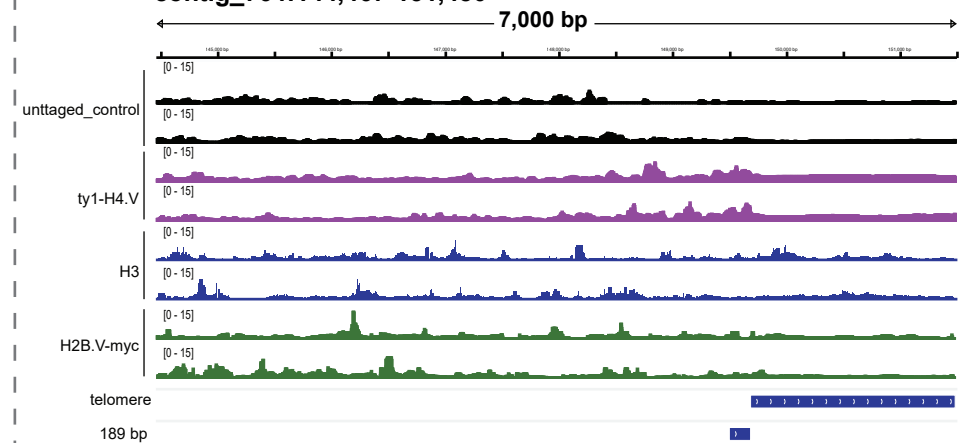

Supplement: S6 Fig — The telomeric and subtelomeric regions were highlighted. The bed tracks represent the telomeric repeats, the 189-bp and when annotated, the trans-sialidases, RHSs, mucins and DGF-1. (PDF) [file ppat.1013310.s006.pdf]

A.

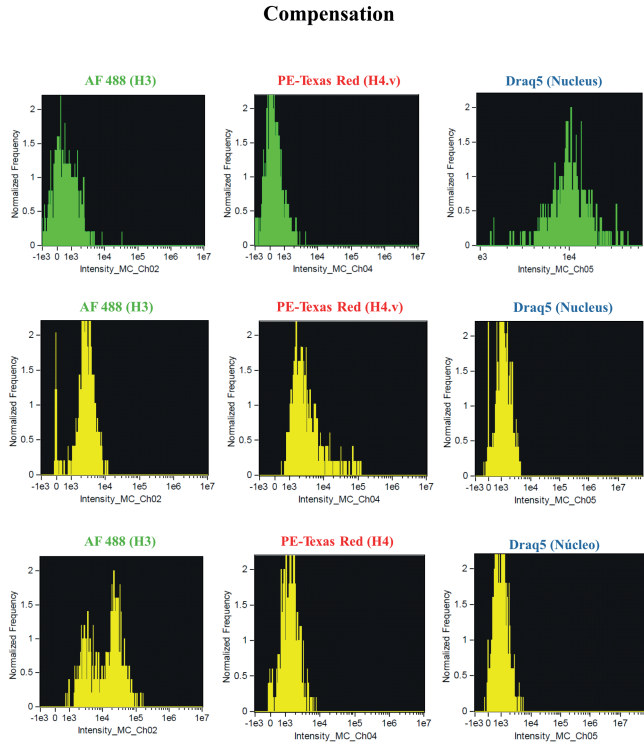

B.

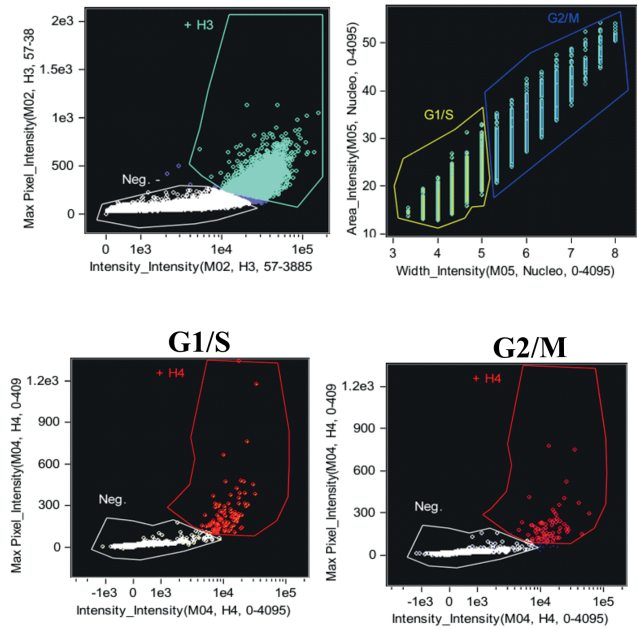

C.

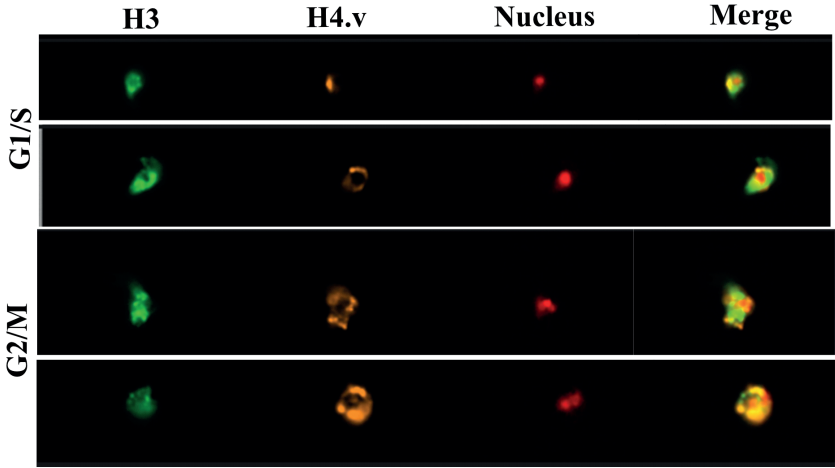

D.

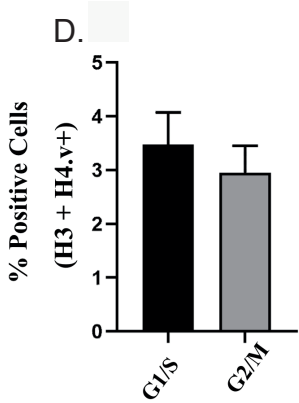

Supplement: S7 Fig — A. Fluorescence intensity plots showing the following labels: Alexa Fluor 488 (histone H3) in channel 02 (ch02); DRAQ5 (nucleus) in channel 05 (ch05), PE-Texas Red (histone H4.V) in channel 04 (ch04). B. Sequential gating strategy to extract H4.V abundance on different cell cycle phases. Initial gate selection of histone H3-positive cells based on “Max Pixel Intensity” versus “Intensity of H3” (top left); followed by cell cycle phase discrimination (G1/S and G2/M) based on nuclear Area Intensity versus Width Intensity (DRAQ5 detection) combined with kinetoplast/flagella quantification (top right); subsequent selection of ty1-H4.V-positive cells (“Max Pixel Intensity” versus “Intensity”) within the H3-positive populations in each cell cycle phase (bottom quadrants). C. Selected images of triple-stained epimastigotes labelled for histone H3, histone ty1-H4.V, and nuclear staining, with corresponding merged images across cell cycle phases obtained from IFC. D. Graph illustrating the percentage of positive cells for both H3 and H4.V in each cell cycle phase. Graphs and images were generated using the Idea 6.3 analysis software (Amnis Corp.). (PDF) [file ppat.1013310.s007.pdf]

A.

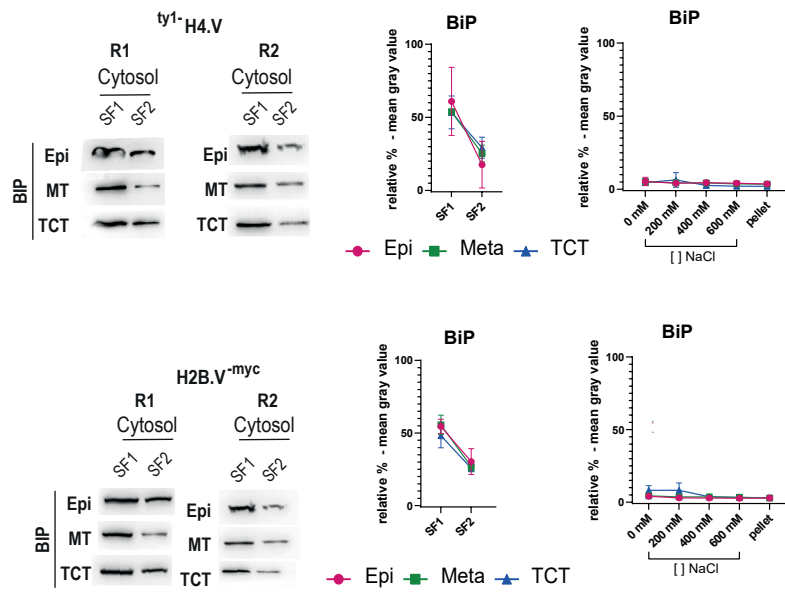

B.

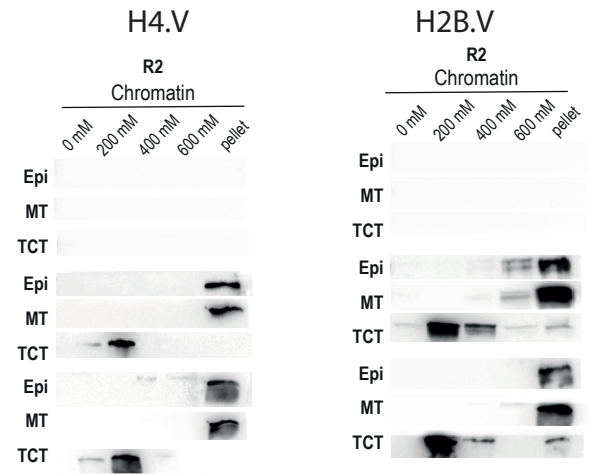

C.

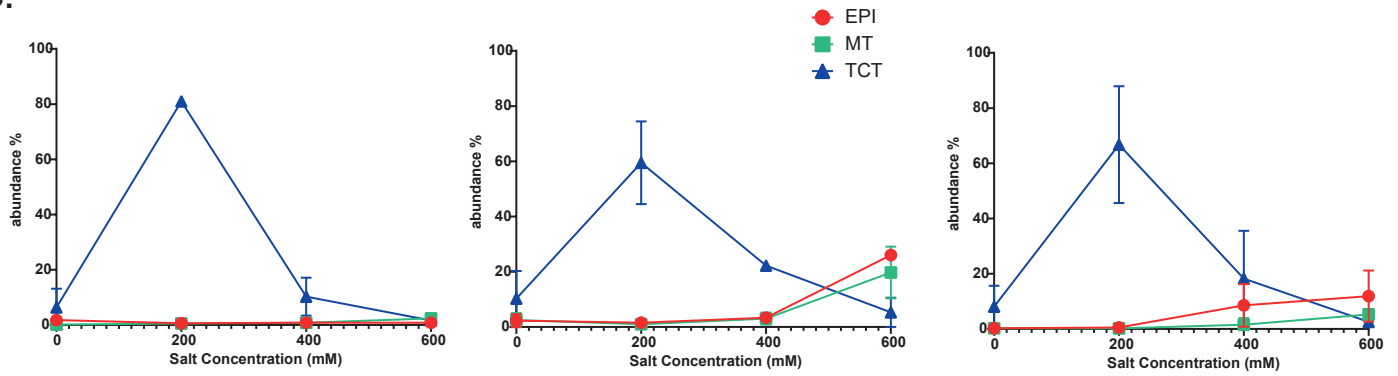

Supplement: S8 Fig — S1 and S2 Figs, soluble fractions from both cytosol and nucleus, from parasites ty1-H4.V and H2B.V-myc were fractionated by SDS-PAGE and evaluated by WB using antibodies against BiP. B. WB analysis analysis of an independent second biological replicate corresponding to the experiment shown in Fig 6. C. Quantification of WB signal intensity (mean gray value with background subtraction) combining both biological replicates. From left to right: H4.V, H2B.V and H3. To enable comparison across life forms and salt conditions, the sum of all mean gray value of each antibody signal in a given life form was normalized to 100%. (PDF) [file ppat.1013310.s008.pdf]
