# Supplementary material for: New Histone H4 variant and H2B variant Exhibits distinct genomic distributions, chromatin affinities, and dynamics throughout life and cell cycle of Trypanosoma cruzi
Source: PLoS Pathog. 2026 Mar 31;22(3):e1013310. doi: 10.1371/journal.ppat.1013310 (PMC13052989; doi:10.1371/journal.ppat.1013310)
Supplement: S2 Fig — A.Scheme representing the H4.V locus before (top) and after (bottom) gene editing by CRISPR. Alignment of the upstream coding regions of the H4.V and H4 loci, showing the primer (H4V_Nterminus_146) used to generate the sgRNA.The donor DNA has three copies of ty-1 sequences; the 5’ UTR of T. brucei actin, 3’UTR of aldolase and the 5’ UTR of aldolase (dark grey); the blasticidin resistance gene (red); and homology arm (Harm-dark blue) that are homologous to the 5’ UTR of H4.V. The red arrows indicate the primers that were used to validate the genome edition, and black arrows indicate the gene transcriptional direction. IGR - IGR represents the intergenic region, and H-Arm represents the homologous arm. B. PCRs of ty1-H4.V parasites using the indicate pair of primers. Amplicons were fractionated by 1% agarose gel showing the insertion of the t ty1 sequences specifically at the H4.V locus. Primers were designed to anneal specifically to the blasticidin-resistance gene (Primer 1), the ty1 sequence (Primer 2), and the H4.V sequence (Primers 3, 4, and 5).Primer 5 was designed to anneal specifically to the H4.V sequence, which differs from canonical H4 by three nucleotides, including at the most distal 3′ end. The two terminal 3′ nucleotides of Primer 3 are unique to H4.V, ensuring specific amplification compared to canonical H4.The genomic DNA of parasites expressing Cas9 were used as negative control (C-). Results do not allow us to infer whether one or both alleles were edited. C. Western blot assay using anti-ty1 confirmed the H4.V expression in epimastigotes clones. Non-transfected parasites were used as negative control (Ctl). D. Parasites expressing Cas9 and ty1-H4.V growth curves (log10). Unpaired T-test of area under the curve, p = ns. E. Metacyclic trypomastigotes expressing Cas9 or Ty1-H4.V were quantified in RPMI culture supernatants after 8 days. Unpaired T-test, p = ns (p = 0.1609). The experiments were performed in biological triplicates. ns: no statistically [file ppat.1013310.s002.pdf]

SF2

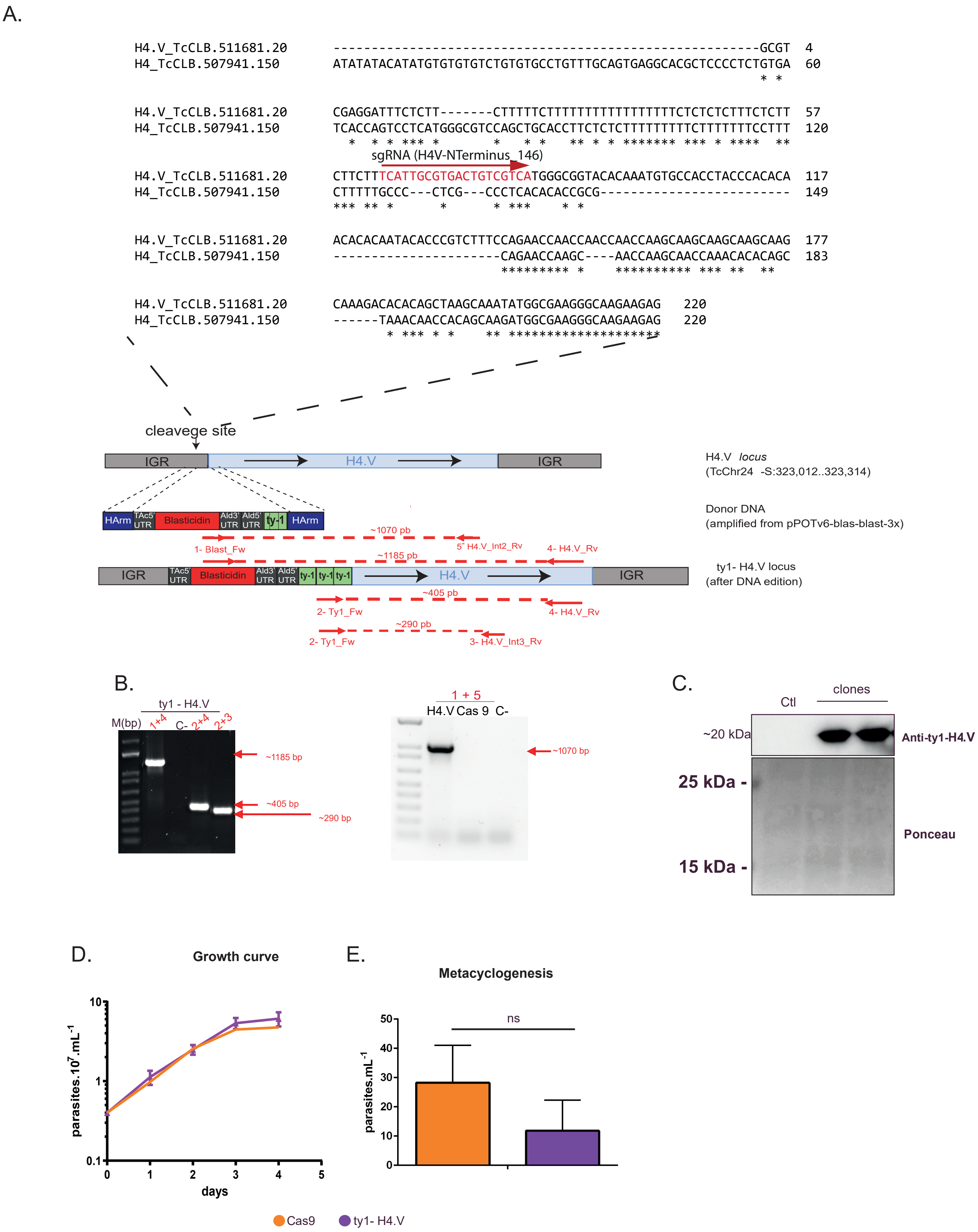
A.
H4.V_TcCLB.511681.20 --GCGT 4
H4_TcCLB.507941.150 ATATATACATATGTGTGTGTCTGTGTGCCTGTTTGCAGTGAGGCACGCTCCCCTCTGTGA 60
H4.V_TcCLB.511681.20 CGAGGATTTCTCTT-------CTTTTTCTTTTTTTTTTTTTTTTTTCTCTCTCTTTCTCTT 57
H4_TcCLB.507941.150 TCACCAGTCCTCATGGGCGTCCAGCTGCACCTTCTCTCTTTTTTTTTCTTTTTTTCCTTT 120
sgRNA (H4V-NTerminus_146)
H4.V_TcCLB.511681.20 CTTCTTTCATTGCGTGACTGTCGTCATGGGCGGTACACAAATGTGCCACCTACCCACACA 117
H4_TcCLB.507941.150 CTTTTTGCCC---CTCG---CCCTCACACACCGCG------------------------ 149
H4.V_TcCLB.511681.20 ACACACAATACACCCGTCTTTCCAGAACCAACCAACCAACCAAGCAAGCAAGCAAGCAAG 177
H4_TcCLB.507941.150 ----------------------CAGAACCAAGC----AACCAAGCAACCAAACACACAGC 183
H4.V_TcCLB.511681.20 CAAAGACACACAGCTAAGCAAATATGGCGAAGGGCAAGAAGAG 220
H4_TcCLB.507941.150 ------TAAACAACCACAGCAAGATGGCGAAGGGCAAGAAGAG 220
cleavege site
IGR
H4.V
IGR
H4.V locus
(TcChr24 -S:323,012..323,314)
HArm
TAc5' UTR
Blasticidin
Ald3' UTR
Ald5' UTR
ty-1
HArm
Donor DNA
(amplified from pPOTv6-blas-blast-3x)
~1070 pb
1- Blast_Fw
5' H4.V_Int2_Rv
4- H4.V_Rv
~1185 pb
IGR
TAc5' UTR
Blasticidin
Ald3' UTR
Ald5' UTR
ty-1
ty-1
ty-1
H4.V
IGR
ty1- H4.V locus
(after DNA edition)
~405 pb
2- Ty1_Fw
4- H4.V_Rv
~290 pb
2- Ty1_Fw
3- H4.V_Int3_Rv
B.
ty1 - H4.V
M(bp)
1+4
C-
2+4
2+3
~1185 bp
~405 bp
~290 bp
1 + 5
H4.V Cas 9 C-
~1070 bp
C.
Ctl
clones
~20 kDa
Anti-ty1-H4.V
25 kDa -
15 kDa -
Ponceau
D.
Growth curve
parasites.10^7.mL^-1
10
1
0.1
0
1
2
3
4
5
days
E.
Metacyclogenesis
ns
parasites.mL^-1
50
40
30
20
10
0
Cas9
ty1- H4.V
